# Violence exposure among adolescent psychiatric patients in Nepal: Socio-demographic and familial risk factors

**DOI:** 10.1371/journal.pone.0355947

**Published:** 2026-08-14

**Authors:** Rampukar Sah, Per Håkan Brøndbo, Bjørn Helge Handegård, Jasmine Ma, Ketil Lenert Hansen, Narmada Devkota, Anne Cecilie Javo

**Affiliations:** 1 Department of Psychology, Faculty of Health Sciences, UiT-The Arctic University of Norway, Tromsø, Norway; 2 CWIN-Nepal CAPMH Research and Outreach Center- Lincoln Marg, Kathmandu, Nepal; 3 Child & Adolescent Psychiatry Unit, Kanti Children’s Hospital, Kathmandu, Nepal; 4 Faculty of Health Sciences, Regional Centre for Child and Youth Mental Health and Child Welfare - North, UiT-The Arctic University of Norway, Tromsø, Norway; 5 CWIN-Nepal, Concerned Centre - Lincoln Marg, Kathmandu, Nepal; 6 Adolescent Mental Health Unit, Mental Hospital, Lagankhel, Patan, Nepal; 7 SANKS - Sámi Klinihkka, Finnmark Hospital Trust, Karasjok, Norway; Patan Academy of Health Sciences, NEPAL

## Abstract

**Background:**

Violence exposure is a major public health concern among adolescents. Yet, evidence on socio-demographic and familial risk factors in adolescent psychiatric populations in Nepal and other low- and middle-income countries is limited.

**Objectives:**

To identify risk factors associated with different forms of violence exposure among adolescents attending a child and adolescent psychiatric unit in Nepal.

**Methods:**

A total of 810 adolescents aged 11–15 years (392 boys, 418 girls) at Kanti Children’s Hospital in Kathmandu, were included. Adolescents reported past-year exposure to violence using adapted PedHITSS and ICAST-C items, while parents provided socio-demographic and family data. Associations were examined using ordinal and logistic regression analyses in SPSS 29.

**Results:**

Physical abuse was more frequent among adolescents from the Dalit caste (OR 1.86, 95% CI 1.03–3.34, p = 0.04), whereas neglect and domestic violence were more frequent among Indigenous/minority groups compared with Brahmin/Chhetri castes (OR 1.72, 95% CI 1.17–2.52, p = 0.006; OR 1.63, 95% CI 1.17–2.27, p = 0.004). Compared with urban areas, emotional abuse was less frequent in rural (OR 0.52, 95% CI 0.34–0.80, p = 0.003) and semi-urban areas (OR 0.60, 95% CI 0.41–0.85, p = 0.005), while sexual abuse and neglect were less frequent in semi-urban areas (OR 0.40, 95% CI 0.21–0.77, p = 0.01; OR 0.35, 95% CI 0.20–0.60, p < 0.001). Emotional abuse, sexual abuse, and neglect were more frequent among females (OR 1.59, 95% CI 1.19–2.13, p = 0.002; OR 3.70, 95% CI 2.27–6.06, p < 0.001; OR 1.75, 95% CI 1.22–2.50, p = 0.002). Increasing maternal age was associated with lower odds of emotional abuse (OR 0.97, 95% CI 0.95–1.00, p = 0.02). Emotional abuse and domestic violence were more frequent in single-parent families compared with extended families (OR 1.98, 95% CI 1.04–3.78, p = 0.04; OR 2.84, 95% CI 1.04–7.78, p = 0.001), and parental illiteracy was linked to increased domestic violence compared with university-level education (OR 2.93, 95% CI 1.15–7.49, p = 0.03).

**Conclusion:**

Violence exposure was more frequent among Dalit and Indigenous/minority groups, females, urban residents, was associated with younger maternal age, single-parent households, and parental illiteracy reflecting need for culturally informed, trauma-focused prevention and intervention.

## Introduction

Violence exposure among children and adolescents is an alarming global public health concern with far-reaching implications for mental health, emotional development, and social integration [1]. Violence exposure encompasses a broad range of experiences, including child maltreatment (physical, emotional, and sexual abuse and neglect), witnessing domestic or community violence, peer victimization and bullying, and other forms of interpersonal violence [1]. Exposure to violence can severely disrupt developmental trajectories, elevate psychiatric morbidity, and impair future well-being [2,3]. The long-term psychological consequences of childhood adversity have been well-documented, often resulting in lasting emotional and cognitive impairments [4–6].

Among these, child maltreatment is one of the most prevalent and well-documented forms of violence exposure. According to WHO estimates based on retrospective reports from adults, the global lifetime prevalence of child maltreatment is 23–28% for physical abuse, around 36% for emotional abuse, and around 18% for sexual abuse. [1]. However, these global averages may obscure substantial disparities between low- and middle-income countries (LMICs) and high-income countries (HICs). In Nepal, this burden is markedly elevated, with 81–83% of children subjected to violent discipline and nearly 50% experiencing physical maltreatment [7,8]. In LMICs such as Nepal, the convergence of entrenched socio-cultural norms, poverty, and social inequality amplifies adolescents’ exposure to multiple forms of violence and poor mental health [9]. Despite increasing global attention, research on the socio-demographic and familial factors that predispose adolescents to violence exposure remains limited, including those in LMICs [10,11]. Studies focusing on clinical populations are even more scarce.

It is further important to note that informants’ conceptualizations of child abuse and neglect are influenced by their sociocultural context. For instance, in Nepal, norms surrounding child-rearing, discipline, and family structure are often shaped by extended family systems, caste hierarchies, and gender roles, which may affect how maltreatment is perceived, experienced, and reported. Practices such as physical and emotional punishment may be more socially accepted in some settings, while forms of emotional abuse or neglect may be underrecognized [12].

Regarding the different risk factors for child maltreatment, international literature shows that patriarchal norms contribute to a higher risk of emotional and sexual abuse for girls, while also discouraging boys from reporting abuse due to stigma surrounding victimhood and masculinity [10,13,14]. Family structure, such as single-parent households and parental mental illnesses may be additional risk factors for child maltreatment [15,16]. Also, low maternal age, which is often the case in LMICs, including Nepal, is linked to increased rates of child abuse and neglect [15–17]. Economic disparities and poverty further compound these risks [18,19]. Children from families with lower parental education are more likely to experience neglect, harsh discipline, or forced labor practices, which are often normalized in resource-deprived settings [20–22]. Moreover, rapid urbanization has introduced additional psychosocial stressors on families. Urban youth may face heightened risks of emotional and sexual violence due to reduced community surveillance, overcrowding, and increased anonymity [23–25]. Additional risk factors specific to certain countries or regions may also contribute, such as caste discrimination. For instance, in parts of Nepal, caste-based discrimination remains deeply rooted in everyday life and disproportionately affects Dalits, a socially marginalized caste [26–28]. Dalit women face especially high risks of sexual and physical violence, with many cases going unpunished [26–28].

The present study aims to identify potential socio-demographic and family-related risk factors associated with different types of violence exposure among adolescents attending a child- and adolescent psychiatric outpatient clinic in Nepal. Such knowledge is particularly important in a context where mental health resources are scarce, and early intervention systems remain underdeveloped [29].

## Materials and methods

### Study design

This study employed a cross-sectional, quantitative research design using data from a clinical sample of adolescents.

### Study site and population

The study was conducted at the Child and Adolescent Psychiatry Outpatient Unit (CAP-OPD) of Kanti Children’s Hospital, a tertiary referral center in Kathmandu, Nepal. The hospital serves a diverse patient population from all geographic regions, castes and ethnic groups, and from different socioeconomic backgrounds.

### Participants

The study population comprised adolescents aged 11–15 years who visited the hospital unit during the period 15 January 2023–15 January 2024. Only adolescents were selected as informants, given their cognitive and linguistic maturity to reliably complete self-administered screening instruments. Adolescents with diagnoses such as intellectual developmental disorder (IDD), acute psychosis, or any other condition that could compromise their cognitive and linguistic capacity to reliably complete self-administered maltreatment screening instruments, were excluded. Due to current hospital regulations at the study site, which do not permit admission of patients older than 14 years, adolescents aged 15–18 years could not be included. Of 1,590 new patients, 642 were excluded based on diagnoses impairing cooperation. Of the remaining 948 patients, 810 consented to participate, yielding a response rate of 85%. The sample size was primarily determined by the fixed one-year data collection period at the clinic, during which 810 eligible participants were enrolled consecutively.

For the purposes of this study, we categorize participants based on caste and ethnicity as recognized by Nepal’s national census [30]. The following three categories were used: Dalits, Brahmin/Chhetri, and Indigenous/Minorities groups. The term “Dalit” refers to historically socially marginalized caste groups, while “Brahmin/Chhetri” are considered socially dominant caste groups. Caste and ethnicity in Nepal are deeply intertwined with historical and structural inequalities. Dalits have historically faced systemic discrimination, limiting their access to education, healthcare, and economic opportunities. Similarly, Indigenous/Minority groups (’Janajati’) face unique challenges due to geographic isolation and cultural marginalization. These structural barriers may contribute to increased household stress and vulnerability to violence [26–28].

### Procedure

Before data collection, all participating adolescents and parents were informed about the project, both orally and in written form. Written assent was obtained from all adolescents, and written informed consent was obtained from their parents, legal guardians or caregivers. Data was collected using a self-administered, paper-based questionnaire completed by adolescents, while socio-demographic and family data were provided by one parent (either mother or father) and recorded manually by staff. Data collection was carried out by two office secretaries and one research assistant, all of whom received training, supervision, and monitoring from the first author throughout the data collection period. Completing the maltreatment questionnaires took approximately 20–30 minutes. However, adolescents were not given a fixed time limit to complete them. No unexpected incidents (e.g., emotional distress) were observed during the procedure. To assure the validity of the sensitive data provided by the adolescents, the forms were completed by each adolescent alone in a separate, quiet room, prior to a clinical consultation, and with trained secretaries available to answer any queries and to clarify possible misunderstandings. Subsequently, the clinicians responsible reviewed the filled-in forms that the participants brought with them to the consultation room. Data plotting was done manually throughout the collection period.

#### Instruments.

*Screening instruments of child maltreatment*

The screening form for child abuse was filled out by the adolescents, assessing their experiences of various forms of abuse: physical, emotional, and sexual abuse. An additional form asked about child neglect and home environment.

The five questions about abuse were taken from the Pediatric Hurt-Insult-Threaten-Scream-Sex (PedHITSS) screening tool for parents [31]. This 5-item questionnaire was originally designed to detect and prompt provider investigation into child abuse in clinical settings and had been validated against the Conflict Tactics Scale: Parent-Child Version (CTSPC) demonstrating good internal consistency (α = .85) and strong concurrent validity (r_s_ = .70, p < .01) [31]. The adolescent version that was made for the present study was modelled after the PedHITSS parent form, with questions rephrased in the first person. Responses on all types of abuse were given on a 5-point Likert scale (0 = Never, 1 = Rarely, 2 = Sometimes, 3 = Fairly often, 4 = Frequently). PedHITSS for parents contains the following questions: “During the last year, how often would you estimate that an immediate family member did each of the following to the child: (1) Physically hurt him/her; (2) Insult him/her or talk down to him/her; (3) Threaten him/her with physical harm; (4) Scream or curse at him/her; or (5) Force him/her to have sex [31]. The questions were used to construct variables under the categories of physical, emotional and sexual abuse. Physical abuse was assessed with the question (1). Sexual abuse was assessed with the question (5). The emotional abuse variable was constructed using the three questions asking about the different aspects of emotional abuse: (2) insults, (3) threats, and (4) cursing. To create a composite variable of emotional abuse frequency, the highest reported frequency among the three items was prioritized.

An additional questionnaire was used to assess aspects of child neglect and home environment. These questions were taken from the ISPCAN Child Abuse Screening Tool, Children’s Version (ICAST-C), a validated 38-item tool developed by the International Society for the Prevention of Child Abuse and Neglect (ISPCAN) [32]. The ICAST-C is a multi-national, multi-lingual, consensus-based survey instrument that has been tested in many countries [33]. As ISPCAN members, we got permission from the ISPCAN group to use the ICAST-C questions for our study.

All instruments were translated into Nepali and then back-translated by bilingual clinicians and professional translators to ensure accuracy and cultural relevance. The translation of tools was done with the standard procedure suggested by the WHO [34].

*Background Information Questionnaire*

Background information was obtained through a structured written questionnaire administered to the parents. Socio-demographic data included residential areas (rural, semi-urban or urban setting), gender, and caste/ethnicity. Information about the family included parental educational level, which was categorized according to the Nepali school system. Data on parental education were collected from both parents. In households with two parents, the higher level of education was used. Data on parents’ physical and mental health histories were collected for both parents in dichotomous (yes/no) form. If either parent had a chronic condition, the variable was coded as “yes.” Other family variables included family structure (classified as single-parent, nuclear, or extended), mother’s age, and monthly income. Income was assessed using a modified version of the Kuppuswamy scale, adapted to Nepal-specific income brackets ranging from ≤ NPR 4,850 to ≥ NPR 97,451 [35].

### Ethics statement and ethical considerations

Ethical approval was obtained from the Institutional Review Committee of the hospital (Reference No: 621) and the Nepal Health Research Council (NHRC) (Reference No: 1227) [36]. All participants received both verbal and written information about the study in the Nepali language, including details on confidentiality, voluntary participation, and the right to withdraw at any time before giving their written consent. Data was anonymized to protect participant’s privacy and stored according to the rules set by the hospital and the NHRC.

The study adhered to the ethical principle of non-maleficence, ensuring that no harm was done to any participant. Clinicians responsible for each patient reviewed all completed forms. Any cases of abuse identified through the screening process that required clinical intervention or legal action, were promptly managed by the attending clinicians in accordance with established institutional protocols, and in coordination with the principal researcher. Thus, adolescents who needed future help were referred to the One Stop Crisis Management Center (OCMC) at Kanti Children’s Hospital and, where deemed appropriate, to the Child Workers in Nepal Concerned Centre (CWIN-Nepal) helpline, protection services and other needed services. This approach ensured participant safety while maintaining the integrity of the research procedures.

### Statistical analysis

Data was analyzed using IBM SPSS version 29.0. Prevalence rates of abuse, neglect and domestic violence were calculated using frequency distributions. Ordinal and logistic regression analyses were conducted to explore associations between different forms of violence and socio-demographic or family-related factors. The overall significance of each ordinal or logistic regression model was assessed using a likelihood ratio test, comparing the fit of the full model against a null model. The critical assumption of proportional odds in ordinal regression was tested for each model using a likelihood ratio test comparing the ordinal model to a generalized (non-proportional) model. For the neglect variable, this assumption was violated; therefore, the outcome was dichotomized into two categories (“Yes” and “No”) and analyzed using logistic regression. For the variables measuring physical, emotional, sexual, and domestic violence, responses were recoded: “Rarely”, “Sometimes” and “Fairly often” were grouped into a single category, while “Never” and “Frequently” were retained as distinct categories. This decision was made to increase cell sizes, improve the stability and precision of the parameter estimates, reduce the risk of overfitting, and facilitate reliable estimation of the proportional odds models. Our regression models included nine predictor variables (15 estimated parameters, excluding threshold parameters). Events-per-parameter (EPP) criterion for sample size adequacy states that approximately 10 events per estimated parameter is the conventional recommendation [37]. EPP values below this threshold may reduce the precision and stability of odds ratio estimates and increase the risk of overfitting [38]. In the present study, the lowest cell counts across the cumulative splits were 83 cases for physical abuse, 32 for sexual abuse, 148 for emotional abuse, 173 for neglect, and 66 for domestic violence, corresponding to EPP values ranging from approximately 2.1 to 11.5.

#### Sample adequacy.

The sample size was primarily determined by the fixed one-year data collection period at the clinic, during which 810 eligible participants were enrolled consecutively. Of the 810 participants enrolled in the study, 31 were excluded due to missing background data, and an additional 65 participants were excluded from the predictor analyses due to their classification in the “other” ethnicity category, which comprised a culturally heterogeneous group. This exclusion reduced model complexity and allowed for a more clearly defined ethnicity variable. Consequently, the predictor analyses were conducted on a final analytical sample of 714 participants.

All statistical tests were conducted using a significance level of 0.05.

## Results

### Distribution of socio-demographic and family-related predictor variables included in the regression analyses

Table 1 presents the distribution of the socio-demographic and family-related predictor variables among the 714 participants included in the regression analyses. As shown in the table, the largest proportion of adolescents resided in urban areas (63.7%), followed by semi-urban (22.1%) and rural (14.1%) regions. The sample was nearly evenly split by gender, with female participants comprising a slight majority (51.0%). Regarding family structure, nuclear families were most common (48.0%), followed closely by extended families (45.7%), while single-parent households were the least frequent (5.6%). A significant minority reported parental physical illnesses (30.1%) and mental illness (12.5%). Most households had secondary-level education as its maximum (48.5%), followed by households with at least one university-educated parent (36.6%).

**Table 1:** Distribution of socio-demographic and family-related predictor variables included in the regression analyses (N = 714).

Variables	N	%
**Residential area**		
Rural	101	14.1
Semi-urban	158	22.1
Urban	455	63.7
**Gender**		
Male	350	49.0
Female	364	51.0
**Family structure**		
Single Parenthood	40	5.0
Nuclear Family	348	48.0
Extended family	326	45.7
**Parent physical illness**		
Yes	215	30.1
No	499	69.9
**Parent mental illness**		
Yes	89	12.5
No	625	87.5
Parental education*		
Illiterate	19	2.7
Primary level	88	12.3
Secondary level	346	48.5
University level	261	36.6
Income**		
Lower class	256	35.9
Middle class	272	38.1
Upper class	186	26.1
Caste and ethnicity***		
Brahmin/Chhetri	453	63.4
Dalits	46	6.4
Indigenous/Minorities group (“Janajati”)	215	30.1

*In the households with two parents, the higher education level was used. Primary level education consists of 5 years of education from grade 1–5. Secondary level education consists of 12 years of education up to grade 12. University level education includes 13 years of education and above.

**World Bank. Updated Income Classification was used.

*** Government’s official classification systems were used for the classification of castes/ethnicity and income.

Most families were classified as either middle-income (38.1%) or low-income (35.9%) households. Brahmin and Chhetri families predominated (63.4%), with smaller representation from Indigenous/Minority families (30.1%) and Dalit families (6.4%). The mean age of mothers was 38 years.

### Distribution of violence exposure outcome variables included in the regression analyses

Table 2 shows the distribution of violence exposure outcomes (physical abuse, emotional abuse, sexual abuse, neglect and domestic violence) included in the regression analysis. For a more detailed description of prevalence rates using all five categories of frequency and the total sample of 810, see our previous paper [39].

**Table 2:** Distribution of violence exposure outcome variables included in the regression analyses (N = 714).

Forms of Violence	N	%
**Physical abuse**		
Never	413	57.8
Rarely – Fairly often	218	30.5
Frequently	83	11.6
**Emotional abuse**		
Never	186	26.1
Rarely – Fairly often	380	53.2
Frequently	148	20.7
**Sexual abuse**		
Never	606	84.9
Rarely – Fairly often	76	10.6
Frequently	32	4.5
**Neglect**		
No	551	75.8
Yes	173	24.2
**Domestic violence**		
Never	435	60.9
Rarely – Fairly often	213	29.8
Frequently	66	9.2

**Note:** Rarely (a couple of times a year), Fairly often (1–2 or more times a month) and Frequently (weekly or more).

Physical Abuse: More than half of the participants (57.8%) reported never having experienced physical abuse. A substantial portion of the sample (30.5%) reported experiencing physical abuse at a frequency ranging from “Rarely” to “Fairly often.” Furthermore, 11.6% of participants reported experiencing physical abuse frequently.
Emotional Abuse: The distribution of emotional abuse shows that a quarter of the participants (26.1%) reported never experiencing it. However, most participants (53.2%) fell into the category of experiencing emotional abuse from “Rarely” to “Fairly often.” Additionally, one in five participants (20.7%) reported experiencing emotional abuse frequently.
Sexual Abuse: Most participants (84.9%) reported never having experienced sexual abuse. Of the remaining participants, 10.6% reported experiencing sexual abuse from “Rarely” to “Fairly often,” while a smaller segment (4.5%) reported experiencing it frequently.
Neglect: Most participants (75.8%) reported that they had not experienced neglect. The data indicates that 24.2% of the participants reported having experienced neglect.
Domestic Violence: Most participants (60.9%) reported never witnessing or experiencing domestic violence. Meanwhile, 29.8% reported exposure to domestic violence at a frequency between “Rarely” and “Fairly often.” Nearly one in ten participants (9.2%) reported frequently witnessing or experiencing domestic violence.

### Predictors of physical abuse

Ordinal regression analysis was conducted to identify predictors of physical abuse (Table 3). The analysis indicated that younger maternal age and belonging to the Dalit group were significant risk factors for experiencing higher physical abuse category. Adolescents from the Dalit group had higher odds of being in a higher physical abuse category compared to those from the Brahmin/Chhetri group (OR = 1.86, 95% CI: 1.03–3.34, p = .04). Additionally, each one-year increase in maternal age was associated with decrease in the odds of reporting a higher physical abuse category (OR = 0.96, 95% CI: 0.94–0.99, p = .004).

**Table 3:** Predictors of physical abuse.

Predictors	OR	95% CI	Wald (p-value)
Age of Mother	0.96	(0.94, 0.99)	8.24 (.004)
Residential Area (Ref: Urban)			
Rural	0.75	(0.48, 1.17)	1.66 (.19)
Semi-urban	0.74	(0.51, 1.07)	2.54 (.11)
Gender (Ref: Female)			
Male	1.30	(0.96, 1.75)	2.95 (.08)
Family Structure (Ref: Extended Family)			
Single Parenthood	1.28	(0.67, 2.42)	0.56 (.45)
Nuclear Family	1.01	(0.75, 1.37)	0.00 (.95)
Parental physical illness (Ref: Yes)			
No	1.22	(0.88, 1.70)	1.46 (.22)
Parental mental illness (Ref: Yes)			
No	1.18	(0.75, 1.84)	0.50 (.48)
Parental education (Ref: University level)			
Illiterate	1.11	(0.40, 3.08)	0.04 (.83)
Primary level	1.20	(0.72, 2.00)	0.50 (.48)
Secondary level	1.15	(0.81, 1.63)	0.61 (.43)
Income (Ref: Upper class)			
Lower class	1.10	(0.72, 1.67)	0.19 (.66)
Middle Class	1.46	(0.99, 2.15)	3.61 (.05)
Ethnicity (Ref: Brahmin/Chhetri group)			
Dalits	1.86	(1.03, 3.34)	4.28 (.04)
Indigenous/Minorities group	0.91	(0.65, 1.27)	0.32 (.57)

Note: **OR** = Odds Ratio; **CI** = Confidence Interval (Lower, Upper). The degrees of freedom (df) for each Wald test = 1. The Nagelkerke R-squared value indicated limited explanatory power (.050). There was no significant evidence to suggest a violation of the proportional odds assumption, supporting the appropriateness of using ordinal regression for the analysis.

### Predictors of emotional abuse

The ordinal regression analysis identified several significant factors associated with emotional abuse (Table 4). Younger maternal age was a significant risk factor (OR = 0.97, 95% CI: 0.95–1.00, p = 0.02), with each additional year of maternal age associated with a decrease in the odds of an adolescent being in a higher category of emotional abuse. Adolescents in urban areas had greater odds of experiencing higher levels of emotional abuse compared to their peers in other areas. Using urban areas as the reference group, the odds of being in a higher emotional abuse category were significantly lower for adolescents in rural areas (OR = 0.52, 95% CI: 0.34–0.80, p = 0.003) and semi-urban areas (OR = 0.60, 95% CI: 0.41–0.85, p = 0.005). Male adolescents had lower odds of being in a higher emotional abuse category compared to females (OR = 0.63, 95% CI: 0.47–0.84, p = 0.002). Finally, family structure was a significant risk factor, as adolescents from single-parent families had higher odds of being in a higher category of emotional abuse compared to those from extended families (OR = 1.98, 95% CI: 1.04–3.78, p = 0.04).

**Table 4:** Predictors of emotional abuse.

Predictors	OR	95% CI	Wald (p-value)
Age of Mother	0.97	(0.95, 1.00)	5.27 (.02)
Residential Area (Ref: Urban)			
Rural	0.52	(0.34, 0.80)	8.91 (.003)
Semi-urban	0.60	(0.41, 0.85)	7.91 (.005)
Gender (Ref: Female)			
Male	0.63	(0.47, 0.84)	10.01 (.002)
Family Structure (Ref: Extended Family)			
Single Parenthood	1.98	(1.04, 3.78)	4.34 (.04)
Nuclear Family	1.03	(0.77, 1.38)	0.03 (.85)
Parental physical illness (Ref: Yes)			
No	0.92	(0.67, 1.27)	0.23 (.62)
Parental mental illness (Ref: Yes)			
No	1.47	(0.94, 2.30)	2.93 (.08)
Parental education (Ref: University level)			
Illiterate	0.92	(0.36, 2.38)	0.03 (.86)
Primary level	1.52	(0.92, 2.49)	2.67 (.10)
Secondary level	1.16	(0.83, 1.63)	0.75 (.38)
Income (Ref: Upper class)			
Lower class	0.87	(0.58, 1.30)	0.45 (.50)
Middle Class	1.11	(0.76, 1.61)	0.30 (.58)
Ethnicity (Ref: Brahmin/Chhetri group)			
Dalits	0.96	(0.70, 1.32)	0.05 (.81)
Indigenous/Minorities group	1.04	(0.57, 1.90)	0.02 (.88)

**Notes: OR** = Odds Ratio; **CI** = Confidence Interval (Lower, Upper). The final model was statistically significant, χ²(15) = 41.17, p < .001. Nagelkerke R^3^ = .065. The degrees of freedom (df) for each Wald test = 1. There was no significant evidence to suggest a violation of the proportional odds assumption, supporting the appropriateness of using ordinal regression for the analysis.

### Predictors of sexual abuse

The ordinal regression analysis identified several significant factors associated with sexual abuse (Table 5). The analysis revealed that male adolescents had significantly lower odds of being in a higher sexual abuse category (OR = 0.27, 95% CI: 0.17–0.44, p < 0.001). Using urban areas as the reference group, adolescents residing in semi-urban areas had lower odds of being in a higher category of sexual abuse (OR = 0.40, 95% CI: 0.21–0.77, p = 0.01). Adolescents from nuclear families) had higher odds of being in a higher abuse category compared to those from extended families (OR = 1.90, 95% CI: 1.20–3.00, p = 0.01). Finally, the level of parental education was a significant factor for sexual abuse. Using university-level education as the reference, adolescents whose parents had no more than a primary-level education had higher odds of being in a higher category of sexual abuse (OR = 2.13, 95% CI: 1.09–4.18, p = 0.028).

**Table 5:** Predictors of sexual abuse.

Predictors	OR	95% CI	Wald (p-value)
Age of Mother	0.97	(0.94, 1.01)	2.25 (.13)
Residential Area (Ref: Urban)			
Rural	0.70	(0.37, 1.31)	1.26 (.26)
Semi-urban	0.40	(0.21, 0.77)	7.50 (.01)
Gender (Ref: Female)			
Male	0.27	(0.17, 0.44)	28.02 (<.001)
Family Structure (Ref: Extended Family)			
Single Parenthood	2.34	(0.96, 5.70)	3.53 (.06)
Nuclear Family	1.90	(1.20, 3.00)	7.46 (.01)
Parental physical illness (Ref: Yes)			
No	0.63	(0.38, 1.06)	3.08 (.07)
Parental mental illness (Ref: Yes)			
No	1.86	(1.00, 3.46)	3.84 (.05)
Parental education (Ref: University level)			
Illiterate	1.63	(0.44, 5.99)	0.54 (.46)
Primary level	2.13	(1.09, 4.18)	4.83 (.028)
Secondary level	1.15	(0.68, 1.95)	0.27 (.60)
Income (Ref: Upper class)			
Lower class	0.79	(0.43, 1.45)	0.58 (.45)
Middle Class	1.05	(0.60, 1.83)	0.03 (.86)
Ethnicity (Ref: Brahmin/Chhetri)			
Dalits	1.51	(0.70, 3.26)	1.10 (.29)
Indigenous/Minorities group	0.86	(0.53, 1.41)	0.34 (.56)

**Notes: OR** = Odds Ratio; **CI** = Confidence Interval (Lower, Upper). The Nagelkerke R-squared value indicated modest explanatory power (.141). The degrees of freedom (df) for each Wald test = 1. There was no significant evidence to suggest a violation of the proportional odds assumption, supporting the appropriateness of using ordinal regression for the analysis.

### Predictors of child neglect

Logistic regression analysis identified significant risk factors for neglect (Table 6). Adolescents residing in semi-urban areas had lower odds of experiencing neglect compared to those in urban areas (OR = 0.35, 95% CI: 0.20–0.60, p < 0.001). Male adolescents had significantly lower odds of neglect compared to female adolescents (OR = 0.57, 95% CI: 0.40–0.82, p = 0.002). Regarding ethnicity, adolescents from Indigenous/Minorities groups had higher odds of neglect compared to those from the Brahmin/Chhetri group (OR = 1.72, 95% CI: 1.17–2.52, p = 0.006).

**Table 6:** Predictors of child neglect.

Variable	OR	95% CI	Wald (p-value)
Age of mother	1.00	(0.97, 1.03)	0.03 (0.87)
Residential Area (Ref: Urban)			
Rural	0.99	(0.59, 1.65)	0.00 (0.97)
Semi-urban	0.35	(0.20, 0.60)	14.20 (< 0.001)
Gender (Ref: Female)			
Male	0.57	(0.40, 0.82)	9.06 (0.002)
Family structure (Ref: Extended family)			
Single Parenthood	1.40	(0.66, 2.98)	0.77 (0.38)
Nuclear	1.14	(0.79, 1.66)	0.51 (0.48)
Parental physical illness			
Yes	0.87	(0.58, 1.30)	0.50 (0.48)
Parental mental illness			
Yes	1.29	(0.76, 2.20)	0.89 (0.35)
Parental education (Ref: University)			
Illiterate	1.67	(0.57, 4.89)	0.86 (0.36)
Primary level	1.06	(0.58, 1.94)	0.04 (0.84)
Secondary level	0.98	(0.64, 1.50)	0.01 (0.92)
Income (Ref: Upper class)			
Lower class	0.99	(0.61, 1.62)	0.01 (0.97)
Middle class	0.81	(0.51, 1.29)	0.80 (0.37)
Ethnicity (Ref: Brahmin/Chhetri group)			
Dalits	1.58	(0.78, 3.18)	1.62 (0.20)
Indigenous/Minorities group	1.72	(1.17, 2.52)	7.58 (0.006)

**Note: OR** = Odds Ratio; **CI** = Confidence Interval (Lower, Upper). Nagelkerke R² = .087).

### Predictors of domestic violence

The ordinal regression analysis identified several significant factors associated with domestic violence (Table 7). Family structure was a significant predictor of domestic violence. Using extended families as the reference group, adolescents from single-parent families had higher odds of being in a higher category of domestic violence (OR = 2.84, 95% CI: 1.50–5.37, p = 0.001). Parental education was also a significant predictor. Using university-level education as the reference group, adolescents from illiterate families had higher odds of being in a higher category of domestic violence (OR = 2.93, 95% CI: 1.15–7.49, p = 0.03). Furthermore, adolescent ethnic background was associated with the outcome. When using the Brahmin/Chhetri group as the reference group, adolescents from the Indigenous/Minorities group had higher odds of being in a higher category of domestic violence (OR = 1.63, 95% CI: 1.17–2.27, p = 0.004).

**Table 7:** Predictors of domestic violence.

Predictor	OR	95% CI	Wald (p-value)
Age of mother	0.98	(0.95, 1.01)	2.52 (.11)
Residential Area (ref: Urban)			
Rural	0.77	(0.49, 1.22)	1.22 (.27)
Semi-urban	0.73	(0.49, 1.08)	2.48 (.12)
Gender (ref: Female)			
Male	0.74	(0.55, 1.00)	3.67 (.06)
Family structure (ref: extended)			
Single parenthood	2.84	(1.50, 5.37)	10.31 (.001)
Nuclear Family	1.27	(0.93, 1.74)	2.26 (.13)
Parental medical illness (ref: Yes)			
No	1.20	(0.86, 1.68)	1.12 (.29)
Parental mental illness (ref: Yes)			
No	1.51	(0.96, 2.37)	3.22 (.07)
Parental education (ref: University level)			
Illiterate	2.93	(1.15, 7.49)	5.05 (.03)
Primary level	1.10	(0.65, 1.85)	0.12 (.73)
Secondary level	1.01	(0.70, 1.45)	0.00 (.96)
Income (ref: Upper class)			
Lower class	0.88	(0.58, 1.34)	0.35 (.56)
Middle Class	0.78	(0.53, 1.16)	1.54 (.21)
Ethnicity (Ref: Brahmin/Chhetri group)			
Dalits	1.38	(0.75, 2.55)	1.08 (.30)
Indigenous/Minorities group	1.63	(1.17, 2.27)	8.38 (.004)

**Notes: OR** = Odds Ratio; **CI** = Confidence Interval (Lower, Upper). The Nagelkerke R-squared value indicated modest explanatory power (.067). The degrees of freedom (df) for each Wald test = 1. There was no significant evidence to suggest a violation of the proportional odds assumption, supporting the appropriateness of using ordinal regression for the analysis.

## Discussion

This study identified multiple socio-demographic and familial risk factors associated with various forms of violence exposure in a clinical sample of adolescents in Nepal.

### Caste/ethnicity

Specific forms of violence exposure were more frequent in marginalized ethnic and caste groups compared to the dominant Brahmin/Chhetri group. These findings align with prior research from Nepal in the general population documenting a disproportionate burden of violence exposure among socially marginalized groups [7,28]. Longstanding structural inequalities such as caste-based discrimination, poverty, and limited access to education and healthcare may increase household stress leading to increased violence [27,28]. In Nepal, socially marginalized communities such as Dalits and Indigenous groups also face multiple barriers to accessing child protection services. These include structural obstacles like geographic remoteness and limited education, as well as social and cultural barriers such as stigma, institutional mistrust, and a lack of culturally and linguistically appropriate services [12,26,27,40]. Together, these constraints may delay detection and reporting of violence exposure, reduce opportunities for timely intervention, and contribute to the persistence and potential escalation of abuse among already vulnerable groups.

### Gender

Our findings of gendered vulnerabilities were most apparent in the context of sexual abuse and neglect, with girls experiencing significantly higher risks. This pattern is consistent with robust global evidence [41,42] and is further supported by review study findings from Asia [43]. The increased risk of neglect for girls suggests prevailing gender-based discrimination in caregiving and resource allocation, especially in traditional societies like Nepal where sons are often prioritized [12–14,44]. Deep-rooted patriarchal norms and male-dominated social structures had historically privileged men, granting them disproportionate power and perpetuating systemic exploitation and marginalization of women [45].

### Place of residence

The adolescent patients residing in urban areas had significantly higher odds of experiencing emotional and sexual abuse, as well as an increased risk of neglect. These findings align with patterns observed in general population studies from countries such as the United States [46], Nepal [8], and other LMICs [7]. In many LMICs, including Nepal, rapid urbanization has led to overcrowded living conditions, unplanned settlements, and overburdened public infrastructure. Consequently, adolescents in urban areas may face heightened risks of emotional and sexual abuse due to reduced community surveillance, overcrowding, and the increased anonymity of urban life [23–25]. Additionally, in such environments, families may experience heightened economic pressures, which can contribute to elevated levels of parental stress and reduce emotional availability for children [47]. The social dislocation and cultural stress associated with internal migration, even when motivated by seeking better opportunities may constitute a risk factor for increased violence [48].

It should be noted that participants in the present study were adolescent patients from diverse regions and residential settings across the country. However, adolescents from urban areas were more represented, due to relatively easier access to the hospital, whereas those from rural and semi-urban areas were comparatively underrepresented due to logistical and access-related challenges.

### Mother’s age

The present study found that each additional year in mother's age corresponded to a 3% decrease in the odds of being in a higher emotional abuse category and a 4% decrease of being in a higher physical abuse category. Young maternal age is a well-documented risk factor for heightened child violence, a finding consistent across both global and local contexts [15–17]. According to other studies, the practice of child marriage remains entrenched in Nepal despite legal prohibitions against it, affecting 33% of girls before the age of 18 and 8% of girls before the age of 15 [49,50]. This prematurely ends formal education for young girls, limiting their future economic opportunities and trapping them into a cycle of poverty known as a catalyst for family stress and violence [18,19]. Beyond economic strain, younger mothers often face developmental challenges that exacerbate parenting stress. They typically have less parenting experience, greater difficulties with emotional regulation, and have limited access to social support networks. This combination of factors can increase frustration and reduce coping mechanisms, leading to an increased reliance on abusive disciplinary strategies [49]. Young motherhood is not confined to low-income countries; even in high-income nations like Canada and Australia, younger maternal age is linked to a greater reported use of physical punishment [51,52].

### Family structure

Adolescents from single-parent families were at higher risk of experiencing more frequent emotional abuse, sexual abuse, and domestic violence compared to those from extended families. These results echo well-established findings from international studies [53–55], emphasizing the protective role of extended family networks [15,16]. The extended family, including grandparents, aunts, uncles, and other relatives, serves as a vital protective buffer against violence towards children. By offering consistent supervision, emotional support, and shared caregiving responsibilities, it may play a crucial role in shielding adolescents from abuse and neglect [56,57].

In the context of Nepal, the extended family structure is particularly vital for supporting families when parents migrate for work abroad. Labor migration is widespread in Nepal, with many parents compelled to work abroad to meet their families’ basic needs. This often results in prolonged family separation, disrupting traditional child-rearing practices and weakening the emotional and practical support systems within the household [58]. The absence of one or both parents can create a care vacuum, reduce supervision and increase the risk of neglect and violence for children left behind [7,59]. These family-level challenges are further compounded by broader systemic issues, including pervasive poverty and an under-resourced child welfare system that lacks the capacity to provide effective oversight and support [60].

### Parental education level

Adolescents from families with low or no parental education showed higher risk of more frequent sexual abuse and domestic violence. Our findings align with previous studies linking lower parental education to increased vulnerability to child neglect in resource-deprived settings [20–22]. The present study contributes to the growing evidence that low parental education is a risk factor for child violence, likely by limiting awareness of children's rights and increasing vulnerability to stress. This underscores the urgent need to break the intergenerational cycle of disadvantage in Nepal through initiatives that promote parental education and child rights awareness.

### Strengths and limitations

#### Strengths.

There are several strengths of the present study. The sample consisted of adolescents admitted to the Child and Adolescent Psychiatry (CAP) outpatient unit over a 12-month period who met the inclusion criteria, resulting in a total of 714 participating adolescents for this study. While this sample size may be considered limited in terms of generalizability, it reflects a complete, consecutive clinical population within the defined setting and timeframe. As such, the sample may be regarded as representative of adolescents receiving inpatient psychiatric care in this context, which strengthens the internal validity of the study. The chosen population ensured sufficient variability in the violence variables, allowing for robust analysis of association with socio-demographic and familial risk factors. The study also benefits from a comprehensive assessment of multiple forms of abuse, supported by rigorous quantitative analysis. Importantly, it provides policy-relevant insights into high-risk groups, offering valuable guidance for targeted interventions. While the findings align with global research, they also contribute new, context-specific knowledge relevant to a LMIC like Nepal.

#### Limitations.

The main limitation of the present study is the generalizability of its findings. We acknowledge that the findings from a hospital-based CAP-OPD may not be fully generalizable to non-clinical populations or to adolescents in other settings or regions. Moreover, several ordinal logistic regression models had EPP ratios below the conventional recommendation of 10, which may have reduced the precision and stability of odds ratio estimates and increased overfitting risk. Accordingly, these findings should be interpreted with caution. Access to outpatient mental health services in Nepal is influenced by factors such as family socioeconomic status, parental education, and mental health awareness. Adolescents from families with greater resources or higher self-efficacy may be more likely to seek and obtain care, meaning that the present sample may not fully represent all adolescents with mental health problems in the clinical population.

Furthermore, the cross-sectional design restricts the ability to infer causality. Although associations between violence exposure and socio-demographic or familial factors were identified, the directionality or temporal sequence of these relationships could not be determined.

In addition, the reliance on self-reported, quantitative measures introduces the possibility of recalling bias and underreporting, especially given the sensitive nature of topics such as abuse and neglect. Cultural stigma, fear, or discomfort in disclosing such experiences may have led some participants to withhold information, potentially leading to conservative estimates of violence prevalence.

Moreover, measuring violence solely by frequency overlooks critical qualitative aspects such as severity and context, potentially simplifying a complex, multifaceted phenomenon. Another limitation is that some important risk factors that may be related to the individuals, their families, and the adolescents’ surroundings were not measured in our study.

### Future recommendations

Present study provides key preliminary insights for future research and culturally tailored interventions. We recommend further research on the interplay of risk factors for violence among Nepali adolescent psychiatric patients, including caste hierarchies, rigid gender norms, and urban violence with larger sample size. A mixed-methods approach would best capture this complexity. Policymakers should prioritize addressing systemic inequalities and expand mental health services to rural and marginalized communities.

## Conclusion

This study demonstrated that violence exposure among adolescent psychiatric patients in Nepal was associated with a range of socio-demographic and familial factors. Elevated risks were observed among adolescents from socially marginalized castes, indigenous and minority groups, and urban areas, as well as among females. Additional risk factors included younger maternal age, single-parent households, and parental illiteracy. These patterns appear to be embedded within and partly shaped by the broader social, cultural, and structural context of Nepal.

The findings highlight the importance of systematically addressing socio-demographic and familial risk factors in the assessment and treatment of adolescents with a history of violence exposure. Moreover, they underscore the need to develop culturally informed prevention and intervention strategies tailored to at-risk adolescent populations.

## Supporting information

S1 File Raw data file, “Paper II data analysis Plos One.sav”. (SAV)
