# Supplementary material for: Violence exposure among adolescent psychiatric patients in Nepal: Socio-demographic and familial risk factors
Source: PLoS One. 2026 Aug 14;21(8):e0355947. doi: 10.1371/journal.pone.0355947 (PMC13475971; doi:10.1371/journal.pone.0355947)
Supplement: Supplementary file 1 [file pone.0355947.s002.docx]

**Title:** Violence Exposure Among Adolescent Psychiatric Patients in Nepal: Socio-Demographic and Familial Risk Factors

**Short Title:** Risk Factors for violence in Nepali Adolescent Psychiatric Youth

**Keywords:** Violence exposure, Nepal, risk factors, adolescent, clinical sample

**Abstract**

**Background**

Exposure to violence during adolescence is a major public health issue, especially for those already experiencing mental health challenges. Adolescents receiving psychiatric care often report higher levels of violence exposure compared to their peers. While global studies have identified various socio-demographic and family-related risk factors linked to such exposure, there is a significant research gap in low- and middle-income countries like Nepal—particularly among clinically referred adolescents.

**Objectives**

This study aimed to identify socio-demographic and familial risk factors associated with different forms of violence exposure among adolescents receiving care at a hospital-based child and adolescent psychiatric unit in Nepal.

**Methods**

A cross-sectional study design was conducted using, with data collected through structured questionnaires administered to caregivers to obtain socio-demographic and familial information, and to adolescents to assess past-year exposure to violence. Statistical analysis was performed using **IBM SPSS Statistics**, with ordinal and logistic regression models applied to examine the associations between different forms of violence exposure and potential risk factors, including gender, caste/ethnicity, residential setting, family structure, parental education, maternal age, and parental medical history.

Results

The analysis revealed several socio-demographic and familial factors significantly associated with adolescents’ exposure to violence. Adolescents from the Dalit caste were more likely to experience physical abuse compared to their Brahmin/Chhetri peers. Those from Indigenous/Minority (Janajati) groups had increased odds of both neglect and domestic violence.

Female adolescents were at higher risk for emotional abuse, sexual abuse, and neglect. Urban residency was linked to greater likelihood of emotional abuse and neglect.

Family structure influenced risk patterns: adolescents from single-parent households were more likely to experience emotional abuse and domestic violence, whereas those from nuclear families faced higher odds of sexual abuse compared to adolescents from extended families. Lower parental education was associated with increased sexual abuse, and parental illiteracy correlated with higher domestic violence.

Conversely, older maternal age appeared protective, reducing the likelihood of both physical and emotional abuse.

**Conclusions**

This study highlights that adolescents attending psychiatric services in Nepal are differentially exposed to various forms of violence based on their socio-demographic and familial characteristics. The findings emphasize the critical importance of integrating considerations of intersecting vulnerabilities—such as female gender, marginalized caste/ethnic background, urban residence, younger maternal age, and lower parental education—into the design of preventive programs and tailored clinical interventions aimed at mitigating violence exposure in this high-risk population.

**Key Word**: arrange in alphabetic orders

Order of Authors: Add task disturbation.

Financial Disclosure: at least type of find and who is provided fund need to mention. add funding details.

Ethics Statement: The study was approved by the Kanti Children's Hospital Ethical Review Committee

(Reference No: 621) and the Nepal Health Research Council (Reference No: 1227).

**Introduction: adequate information to support the clarify the study**

**Method : Arrange the method part**

1. Research design: This study used a cross-sectional, quantitative research design
2. Setting: give the complete information about research setting
3. Population: clearly mention about research population.
4. Sample Size: How you chooses the sample, and what was the sample distribution.
5. Sampling Technique: clearly mention sampling technique
6. Data collection Procedure: duration of data collection,
7. Statistical Analysis: mention clearly Differential and inferential statistics
8. Result: Socio-demographic data- arrange in order wise variable, Table description mention just below the table.
9. Discussion part: write discussion based on objectives. Like socio-demographic information discussion write under socio-demographic heading, and so on.
10. Conclusion: Before limitation and recommendation write conclusion. Conclusion should be based on research findings. Policy making portion you will recommended.
11. Limitation : Mention point wise
12. Recommendation:
