# Supplementary material for: Violence exposure among adolescent psychiatric patients in Nepal: Socio-demographic and familial risk factors
Source: PLoS One. 2026 Aug 14;21(8):e0355947. doi: 10.1371/journal.pone.0355947 (PMC13475971; doi:10.1371/journal.pone.0355947)
Supplement: Supplementary file 2 [file pone.0355947.s003.docx]

***Violence Exposure Among Adolescent Psychiatric Patients in Nepal: Socio-Demographic and Familial Risk Factors***

Rampukar Sah ^1,2,3*^, Per Håkan Brøndbo^1^, Bjørn Helge Handegård^4^, Jasmine Ma^2^, Ketil Lenert Hansen^4^, Narmada Devkota^2,5^, Anne Cecilie Javo^6^

^1^ Department of Psychology, Faculty of Health Sciences, UiT-The Arctic University of Norway, Hansine Hansens veg 18, 9019 Tromsø, Norway

^2^ CWIN-Nepal CAPMH Research and Outreach Center- Lincoln Marg, Kathmandu 44600, Nepal

^3^ Child & Adolescent Psychiatry Unit, Kanti Children’s Hospital, Kathmandu 44600, Nepal

^4^ Regional Centre for Child and Youth Mental Health and Child Welfare - North, Faculty of Health Sciences, UiT-The Arctic University of Norway, Hansine Hansens veg 18, 9019, Tromsø, Norway

^5^ Adolescent Mental Health Unit, Mental Hospital, Lagankhel, Patan 44700, Nepal

^6^ SANKS - Sámi Klinihkka, Finnmark Hospital Trust, 9730 Karasjok, Norway

Corresponding author E-mail: rampukarsah@yahoo.com

**Short title:** Risk Factors for violence in Nepali Adolescent Psychiatric Youth

**Abstract**

**Background**

Exposure to violence during adolescence is a critical public health concern, particularly among individuals with existing mental health vulnerabilities. Adolescents attending psychiatric services are likely to report elevated levels of violence exposure. International research has identified several socio-demographic and familial factors associated with such exposure. However, in low- and middle-income countries (LMICs) such as Nepal, there is lack of research on these associations, particularly among clinically referred youth.

**Objectives**

This study aimed to identify socio-demographic and familial risk factors associated with various forms of violence exposure among adolescents receiving care at a hospital-based child and adolescent psychiatric unit in Nepal.

**Methods**

A cross-sectional study was conducted using structured questionnaires to collect socio-demographic and familial background information from caregivers and data on past-year violence exposure from adolescent patients. Ordinal and logistic regression analyses were used to examine associations between different types of violence exposure and possible risk factors including gender, caste/ethnicity, residential setting, family structure, parental education, maternal age, and parental medical history.

**Results**

Several socio-demographic and familial risk factors were significantly associated with exposure to violence. Adolescents from the Dalit group (socially marginalized caste) had higher odds of reporting more physical abuse compared to adolescents from the Brahmin/Chhetri group (dominant castes group). Adolescents from the Indigenous/Minorities group (the “Janajati”) had higher odds of both neglect and domestic violence. Female adolescents had significantly higher odds of experiencing emotional abuse, sexual abuse, and neglect. Residing in urban areas was associated with higher odds of emotional abuse and neglect. Compared to extended families, adolescents from single-parent families had higher odds of both emotional abuse and domestic violence, whereas adolescents from nuclear families had higher odds of experiencing sexual abuse. Lower parental education was associated with higher odds of sexual abuse, and illiteracy was associated with domestic violence. Older maternal age was found to be a protective factor for both physical and emotional abuse.

**Conclusions**

This study from Nepal highlights significant associations between different forms of violence experienced by adolescent psychiatric patients and their socio-demographic profiles and familial characteristics. The findings emphasize the importance of addressing intersecting vulnerabilities such as female gender, socially marginalized caste/ethnic status, urban residence, young maternal age and limited family education when designing prevention programs and clinical interventions.

Keywords: Violence exposure, Nepal, risk factors, adolescent, clinical sample

**Introduction**

Violence against adolescents is an alarming global public health concern with far-reaching implications for mental health, emotional development, and social integration [1]. Exposure to violence, whether physical, emotional, sexual, or through neglect, can severely disrupt developmental trajectories, elevate psychiatric morbidity, and impair future well-being [2,3]. The long-term psychological consequences of childhood adversity have been well-documented, often resulting in lasting emotional and cognitive impairments [4-6].

In low- and middle-income countries (LMICs) such as Nepal, the convergence of entrenched socio-cultural norms, poverty, and social inequality amplifies adolescents’ exposure to multiple forms of violence and poor mental health [7]. The intersection of these factors may create significant challenges to the well-being of young people. Despite increasing global attention, research on the socio-demographic and familial factors that predispose adolescents to violence exposure remains limited - particularly in LMICs, including those in South Asia [8,9]. Studies focusing on clinical populations in these regions are even more scarce.

Nepal’s socio-cultural landscape poses unique challenges. Deep-rooted caste hierarchies, gender-based discrimination, and evolving family structures may contribute significantly to adolescents’ vulnerability to violence [10]. International literature generally shows that patriarchal norms contribute to a higher risk of emotional and sexual abuse for girls, while also discouraging boys from reporting abuse due to stigma surrounding victimhood and masculinity [8,11,12].

Family structure, such as single-parent households, and parental mental illnesses may be additional risk factors for adolescent maltreatment [13,14]. Also, low maternal age, which is often the case in LMICs, including Nepal, is linked to increased rates of child abuse and neglect [13-15]. Economic disparities and poverty further compound these risks [16,17]. Adolescents from families with lower parental education are more likely to experience neglect, harsh discipline, or forced labor practices which are often normalized in resource-deprived settings [18-20].

Caste-based discrimination in Nepal is deeply rooted in everyday life, severely affecting Dalit (socially marginalized caste) communities [21-23]. Dalit women face especially high risks of sexual and physical violence, with many cases going unpunished [21-23]. Moreover, rapid urbanization has introduced additional psychosocial stressors on families. Urban adolescents may face heightened risks of emotional and sexual violence due to reduced community surveillance, overcrowding, and increased anonymity [24-26].

The present study aims to identify potential risk factors associated with violence exposure among adolescents attending a child- and adolescent psychiatric outpatient clinic in Nepal. Gaining a deeper understanding of this population is particularly urgent given the limited availability of mental health resources and early intervention systems in the country [27]. This study was guided by the primary research question: *Are specific socio-demographic and family-related risk factors associated with different types of violence exposure?*

**Methods and Materials**

***Study Site and Population***

Nepal, located in South Asia, is a multicultural nation with 142 caste and ethnic groups, the majority of whom are Hindu [28]. As of 2024, its population reached 31 million, with approximately 35% under the age of 15 [29]. Mental health services, particularly for children and adolescents, are limited, especially in remote areas, due to inadequate infrastructure, long distance, and shortage of specialists. The first Child and Adolescent Psychiatric Outpatient Unit (CAP-OPD) was established in 2015 at Kanti Children’s Hospital (KCH), Kathmandu, and has since expanded. It is the only CAP facility in Nepal, with over 20 consultants (psychiatrists, clinical psychologists, and social workers) and accepts children from all over the country for both outpatient- and inpatient psychiatric treatment. This study was conducted among patients attending this hospital’s CAP-OPD services. The study was approved by the Kanti Children's Hospital Ethical Review Committee (Reference No: 621) and the Nepal Health Research Council (Reference No: 1227).

***Participants***

Participants were adolescents aged 11–15 and their parents who visited the CAP-OPD in a 12-month period, from 15/01/23 to 15/01/24. Patients were admitted to the hospital irrespective of their caste or ethnic background. For the purposes of this study, we categorize participants based on caste and ethnicity as recognized by Nepal’s national census. The following three categories were used: Dalits, Brahmin/Chhetri, and Indigenous/Minorities group. The term “Dalit” refers to historically socially marginalized caste groups, while “Brahmin/Chhetri” are considered socially dominant caste groups. These classifications reflect structural positions in Nepali society rather than intrinsic worth or ability. Indigenous/ethnic minorities are officially recognized by Nepal as “Janajati*”* a diverse set of ethnic groups with distinct languages, cultures, and histories. Of 1,590 new patients, 642 were excluded based on diagnoses impairing cooperation (e.g., intellectual developmental disorder, acute psychosis). Of the remaining 948 patients, 810 consented to participate, yielding a response rate of 85%.

***Study Design***

This study employed a cross-sectional, quantitative research design using data from a clinical sample of adolescents.

***Procedure***

Data collection was conducted by two office secretaries and one research assistant. All three were trained and supervised by the first author, who also monitored their work. Adolescents and parents received both verbal and written information about the study in Nepali, and written informed consent was obtained from all the participants. Parents provided socio-demographic and family data. Adolescents completed screening instruments on abuse, neglect, and home environment in a separate room before their clinical consultation at the OPD, with staff available for clarification. The completed forms were checked by the clinicians responsible before they were handed over to the researcher (first author). Data were manually recorded.

***Instruments***

*Screening instruments of child maltreatment*

The screening form for child abuse was filled out by the adolescents, assessing their experiences of various forms of abuse: physical, emotional, and sexual abuse. An additional form asked about child neglect and home environment.

The five questions about the different forms of child abuse were taken from the Pediatric Hurt-Insult-Threaten-Scream-Sex (PedHITSS) screening tool for parents [30]. This 5-item questionnaire was originally designed to detect and prompt provider investigation into child abuse in clinical settings and had been validated against the Conflict Tactics Scale: Parent-Child Version (CTSPC) [30].

The adolescent version that was made for the present study was modelled after the PedHITSS parent form, with questions rephrased in the first person. It contained the following questions: (1) “*During the past year, how often has an adult - either within your family or outside - intentionally hurt you physically?*”; (2) “*During the last year, how often did an adult either within your family or outside - insult or talk down to you?*”; (3) “*During the past year, how often did an adult - either within your family or outside - threaten to physically harm you?*”; (4) “*During the past year, how often did an adult, ether within your family or outside - scream or curse you?*”; (5a) “*Has any adult in your family or outside ever forced you to engage in sexual acts?*” (For example: touching their private parts, or touching your private parts, or having sexual intercourse)?”; (5b) “*If yes, during the past year, how often did it happen to you?*”.

Responses on abuse were given on a 5-point Likert scale (0 = Never, 1 = Rarely (a couple of times a year), 2 = Sometimes (monthly or less), 3 = Fairly often (1-2 or more times a month), 4 = Frequently (weekly or more).

For this study’s analyses, the 5-point Likert scale was reduced by grouping responses on “Rarely”, “Sometimes”, and “Fairly often” into one single category labelled “Rarely-Fairly often”, while “Never” and “Frequently” were retained as distinct categories.

The above questions were used to construct variables under the categories of physical, emotional and sexual abuse. Physical abuse was assessed with the question (1). Sexual abuse was assessed with the question (5b). The emotional abuse variable was constructed using the three questions asking about the different aspects of emotional abuse: (2) insults, (3) threats, and (4) cursing. To create a composite variable of emotional abuse frequency, the highest reported frequency among the three items was prioritized. If a participant answered "Frequently" on any one item, he or she was classified as experiencing emotional abuse “frequently”. Similarly, reporting "Fairly Often" on at least two of the three items also led to a "Frequently" classification. If neither of these conditions was met, the classification was based on either the summed score or the highest individual score across the three items (*insult or talk down to; threaten with physical harm; scream or curse*), consistent with the ordinal scale used for other abuse variables.

An additional questionnaire was used to assess aspects of child neglect and home environment. These questions were taken from the ISPCAN Child Abuse Screening Tool, Children’s Version (ICAST-C), a validated 38-item tool developed by the International Society for the Prevention of Child Abuse and Neglect (ISPCAN) [31]. The ICAST-C is a multi-national, multi-lingual, consensus-based survey instrument that has been tested in many countries [32]. As ISPCAN members, we got permission from the ISPCAN group to use the ICAST-C questions for our study. The question about child neglect was: “*During the last year, did you experience any of the following events at home?”*, with the following 6 statements (options): “You did not get enough to eat (went hungry) and/or drink (were thirsty)”, “You had to wear clothes that were dirty, torn, or inappropriate for the season”, “You were not taken care of when you were sick or injured”, “You were hurt or injured because no adult was supervising”, “You did not feel cared for”, “You were made to feel unimportant”. The overall question about home environment/domestic violence was: “*During the last year, how have you experienced your home life?*” with the following 6 questions: “Do you feel safe in your family?”, “Has anyone in your home used alcohol and/or drugs and then behaved in a way that frightened you?”, “Have you seen adults in your home shouting and screaming in a way that frightened you?”, “Have you seen adults in your home hurt each other physically (e.g., hitting, slapping, or kicking)?”, “Have you seen adults in your home use knives, guns, stick, rocks or other things to hurt or scare someone else inside home?”, “Have you been mistreated or bullied by your brother(s) or sister(s) at home?” Participants could select multiple responses where applicable.

All instruments were translated into Nepali and then back translated by bilingual clinicians and professional translators to ensure accuracy and cultural relevance.

*Background Information Questionnaire*

Background information was collected through a structured written questionnaire administered to the parents. Socio-demographic data included residential areas (rural, semi-urban or urban setting), gender, and caste/ethnicity. Information about the family included parental educational level, which was categorized according to the Nepali school system. Other family variables included family structure (classified as single-parent-, nuclear-, or extended), mother’s age, and monthly income. Income was assessed using a modified version of the Kuppuswamy scale, adapted to Nepal-specific income brackets ranging from ≤ NPR 4,850 to ≥ NPR 97,451 [33]. Parent’s health history documentation included any chronic physical or mental illnesses. Standardized operational definitions were consistently applied, particularly in the classification of family structure and income brackets, which adhered to Nepal's national economic classification systems.

***Ethical Considerations***

Ethical approval was obtained from the Institutional Review Committee of the hospital and the Nepal Health Research Council (NHRC, https://nhrc.gov.np/). All participants received both verbal and written information about the study in the Nepali language, including details on confidentiality, voluntary participation, and the right to withdraw at any time. Data was anonymized to protect participant’s privacy and stored according to the rules set by the hospital and the NHRC.

Any cases of abuse requiring clinical intervention or legal action were managed by the attending clinicians in accordance with institutional protocols.

**Statistical Analysis**

Data was analyzed using IBM SPSS version 29.0. Prevalence rates of abuse, neglect and domestic violence were calculated using frequency distributions. Ordinal and logistic regression analyses were conducted to explore associations between different forms of violence and socio-demographic or family-related factors. The overall significance of each ordinal or logistic regression model was assessed using a likelihood ratio test, comparing the fit of the full model against a null model. The critical assumption of proportional odds in ordinal regression was tested for each model using a likelihood ratio test comparing the ordinal model to a generalized (non-proportional) model. For the neglect variable, this assumption was violated; therefore, the outcome was dichotomized into two categories (“Yes” and “No”) and analyzed using logistic regression. For the variables measuring physical, emotional, sexual, and domestic violence, responses were recoded: “Rarely”, “Sometimes” and “Fairly often” were grouped into a single category, while “Never” and “Frequently” were retained as distinct categories. To enhance the robustness of the analyses and address issues related to sparse data, and to account for categories that were not fully mutually exclusive, reducing the number of categories was deemed necessary. While this approach simplified the analysis, it involved some loss of information about the ordinal nature of the original variables. All statistical tests were conducted using a significance level of 0.05.

**Results**

**Background data**

A description of the selected socio-demographic and family-related variables is presented in Table 1. As shown in the table, the largest proportion of adolescents resided in urban areas (63.7%), followed by semi-urban (22.1%) and rural (14.1%) regions. The sample was nearly evenly split by gender, with female participants comprising a slight majority (51.0%). Regarding family structure, nuclear families were most common (48.0 %), followed closely by extended families (45.7%), while single-parent households were the least frequent (5.6%). A significant minority reported parental chronic illnesses (30.1%). Most parents had completed secondary-level schooling (48.5%), followed by university-educated parents (36.6%). Most families were classified as either middle-income (38.1%) or low-income (35.9%). Brahmin and Chhetri families predominated (63.4%), with smaller representation from Indigenous/Minority families (30.1%) and Dalit families (6.4%). The mean age of mothers was 38 years.

**Table 1: Description of selected familial and socio-demographic variables as reported by the parents**

| **Variables** | **N** | **%** |
| --- | --- | --- |
| **Residential area** |  |  |
| Rural | 101 | 14.1 |
| Semi-urban | 158 | 22.1 |
| Urban | 455 | 63.7 |
| **Gender** |  |  |
| Male | 350 | 49.0 |
| Female | 364 | 51.0 |
| **Family structure** |  |  |
| Single Parenthood | 40 | 5.0 |
| Nuclear Family | 348 | 48.0 |
| Extended family | 326 | 45.7 |
| **Parent physical illness** |  |  |
| Yes | 215 | 30.1 |
| No | 499 | 69.9 |
| **Parent mental illness** |  |  |
| Yes | 89 | 12.5 |
| No | 625 | 87.5 |
| **Parental education*** |  |  |
| Illiterate | 19 | 2.7 |
| Primary level | 88 | 12.3 |
| Secondary level | 346 | 48.5 |
| University level | 261 | 36.6 |
| **Income**** |  |  |
| Lower class | 256 | 35.9 |
| Middle class | 272 | 38.1 |
| Upper class | 186 | 26.1 |
| **Caste and ethnicity***** |  |  |
| Brahmin/Chhetri | 453 | 63.4 |
| Dalits | 46 | 6.4 |
| Indigenous/Minorities group (“Janajati”) | 215 | 30.1 |

**In the households with two parents, the higher education level was used. Primary level education consists of 5 years of education from grade 1 to 5. Secondary level education consists of 12 years of education up to grade 12. University level education includes 13 years of education and above.*

*********World Bank. Updated Income Classification was used.*

*************** *Government’s official classification systems were used for the classification of castes/ethnicity and income*

**Prevalences of different forms of abuse and neglect as reported by the adolescents**

Table 2 shows the prevalence of different types of abuse, neglect and domestic violence during the past 12 months.

Physical Abuse: More than half of the participants (56.2%) reported never having experienced physical abuse. A substantial portion of the sample (31.4%) reported experiencing physical abuse at a frequency ranging from "Rarely" to "Fairly often." Furthermore, 12.5% of participants reported experiencing physical abuse frequently.

Emotional Abuse: The distribution of emotional abuse shows that a quarter of the participants (25.6%) reported never experiencing it. However, most participants (53.8%) fell into the category of experiencing emotional abuse from "Rarely" to "Fairly often." Additionally, one in five participants (20.6%) reported experiencing emotional abuse frequently.

Sexual Abuse: Most participants (84.1%) reported never having experienced sexual abuse. Of the remaining participants, 11.0% reported experiencing sexual abuse from "Rarely" to "Fairly often," while a smaller segment (4.9%) reported experiencing it frequently.

Neglect: Most participants (75.1%) reported that they had not experienced neglect. The data indicates that 24.9% of the participants reported having experienced neglect.

Domestic Violence: Most participants (60.0%) reported never witnessing or experiencing domestic violence. Meanwhile, 30.2% reported exposure to domestic violence at a frequency between "Rarely" and "Fairly often." Nearly one in ten participants (9.8%) reported frequently witnessing or experiencing domestic violence.

For a more detailed description of prevalence rates using all five categories of frequency, see our previous paper [34].

**Table 2: Prevalence of the different forms of violence as reported by the adolescents**

| **Forms of Violence** | **N** | **%** |
| --- | --- | --- |
| **Physical abuse** |  |  |
| Never | 455 | 56.2 |
| Rarely - Fairly often | 254 | 31.4 |
| Frequently | 101 | 12.5 |
| **Emotional abuse** |  |  |
| Never | 207 | 25.6 |
| Rarely - Fairly often | 436 | 53.8 |
| Frequently | 167 | 20.6 |
| **Sexual abuse** |  |  |
| Never | 681 | 84.1 |
| Rarely - Fairly often | 89 | 11.0 |
| Frequently | 40 | 4.9 |
| **Neglect** |  |  |
| No | 608 | 75.1 |
| Yes | 202 | 24.9 |
| **Domestic violence** |  |  |
| Never | 486 | 60.0 |
| Rarely - Fairly often | 245 | 30.2 |
| Frequently | 79 | 9.8 |

**Note:** *Rarely (a couple of times a year), Fairly often (1-2 or more times a month) and Frequently (weekly or more)*

**Predictors of physical abuse**

Ordinal regression analysis was conducted to identify predictors of physical abuse (Table 3). The analysis indicated that younger maternal age and belonging to the Dalit group were significant risk factors for experiencing more physical abuse. Adolescents from the Dalit group had 86% higher odds of being in a higher physical abuse category compared to those from the Brahmin/Chhetri group. Additionally, each one-year increase in maternal age was associated with a 4% decrease in the odds of reporting a higher physical abuse category.

**Table 3: Predictors of Physical abuse**

| Predictors | OR | 95% CI | Wald (p-value) |
| --- | --- | --- | --- |
| Age of Mother | 0.96 | (0.94, 0.99) | 8.24 (.004) |
| Residential Area (Ref: Urban) |  |  |  |
| Rural | 0.75 | (0.48, 1.17) | 1.66 (.19) |
| Semi-urban | 0.74 | (0.51, 1.07) | 2.54 (.11) |
| Gender (Ref: Female) |  |  |  |
| Male | 1.30 | (0.96, 1.75) | 2.95 (.08) |
| Family Structure (Ref: Extended Family) |  |  |  |
| Single Parenthood | 1.28 | (0.67, 2.42) | 0.56 (.45) |
| Nuclear Family | 1.01 | (0.75, 1.37) | 0.00 (.95) |
| Parent physical illness (Ref: Yes) |  |  |  |
| No | 1.22 | (0.88, 1.70) | 1.46 (.22) |
| Parent mental illness (Ref: Yes) |  |  |  |
| No | 1.18 | (0.75, 1.84) | 0.50 (.48) |
| Parental education (Ref: University level) |  |  |  |
| Illiterate | 1.11 | (0.40, 3.08) | 0.04 (.83) |
| Primary level | 1.20 | (0.72, 2.00) | 0.50 (.48) |
| Secondary level | 1.15 | (0.81, 1.63) | 0.61 (.43) |
| Income (Ref: Upper class) |  |  |  |
| Lower class | 1.10 | (0.72, 1.67) | 0.19 (.66) |
| Middle Class | 1.46 | (0.99, 2.15) | 3.61 (.05) |
| Ethnicity (Ref: Brahmin/Chhetri group) |  |  |  |
| Dalits | 1.86 | (1.03, 3.34) | 4.28 (.04) |
| Indigenous/Minorities group | 0.91 | (0.65, 1.27) | 0.32 (.57) |

*Note:* ***OR*** *= Odds Ratio;* ***CI*** *= Confidence Interval (Lower, Upper). The degrees of freedom (df) for each Wald test = 1. The Nagelkerke R-squared value indicated limited explanatory power (.050). There was no significant evidence to suggest a violation of the proportional odds assumption, supporting the appropriateness of using ordinal regression for the analysis.*

**Predictors of Emotional Abuse**

The ordinal regression analysis identified several significant factors associated with emotional abuse. Younger maternal age was a significant risk factor, with each additional year of maternal age associated with a 3% decrease in the odds of an adolescent being in a higher category of emotional. Adolescents residing in urban areas had higher odds of being in a higher emotional abuse category than their peers in other areas. When using urban areas as the reference group, the analysis showed that adolescents in rural areas had 48% lower odds, and those in semi-urban areas had 45% lower odds of being in a higher emotional abuse category. Male adolescents had 37% higher odds of being in a higher emotional abuse category compared to females Finally, family structure was a significant risk factor, as adolescents from single-parent families had 98% higher odds of being in a higher category of emotional abuse compared to those from extended families.

**Table 4. Predictors of Emotional Abuse**

| Predictors | OR | 95% CI | Wald (p-value) |
| --- | --- | --- | --- |
| Age of Mother | 0.97 | (0.95, 1.00) | 5.27 (.02) |
| Residential Area (Ref: Urban) |  |  |  |
| Rural | 0.52 | (0.34, 0.80) | 8.91 (.003) |
| Semi-urban | 0.60 | (0.41, 0.85) | 7.91 (.005) |
| Gender (Ref: Female) |  |  |  |
| Male | 0.63 | (0.47, 0.84) | 10.01 (.002) |
| Family Structure (Ref: Extended Family) |  |  |  |
| Single Parenthood | 1.98 | (1.04, 3.78) | 4.34 (.04) |
| Nuclear Family | 1.03 | (0.77, 1.38) | 0.03 (.85) |
| Parent physical illness (Ref: Yes) |  |  |  |
| No | 0.92 | (0.67, 1.27) | 0.23 (.62) |
| Parent mental illness (Ref: Yes) |  |  |  |
| No | 1.47 | (0.94, 2.30) | 2.93 (.08) |
| Parental education (Ref: University level) |  |  |  |
| Illiterate | 0.92 | (0.36, 2.38) | 0.03 (.86) |
| Primary level | 1.52 | (0.92, 2.49) | 2.67 (.10) |
| Secondary level | 1.16 | (0.83, 1.63) | 0.75 (.38) |
| Income (Ref: Upper class) |  |  |  |
| Lower class | 0.87 | (0.58, 1.30) | 0.45 (.50) |
| Middle Class | 1.11 | (0.76, 1.61) | 0.30 (.58) |
| Ethnicity (Ref: Brahmin/Chhetri group) |  |  |  |
| Dalits | 0.96 | (0.70, 1.32) | 0.05 (.81) |
| Indigenous/Minorities group | 1.04 | (0.57, 1.90) | 0.02 (.88) |

**Notes: *OR*** *= Odds Ratio;* ***CI*** *= Confidence Interval (Lower, Upper). The final model was statistically significant, χ²(15) = 41.17, p < .001. Nagelkerke R² = .065. The degrees of freedom (df) for each Wald test = 1. There was no significant evidence to suggest a violation of the proportional odds assumption, supporting the appropriateness of using ordinal regression for the analysis.*

**Predictors of Sexual Abuse**

The analysis revealed that male adolescents had significantly lower odds of being in a higher abuse category; the odds for males were 73% lower than the odds for females. Using urban areas as the reference group, adolescents residing in semi-urban areas had 60% lower odds of being in a higher category of sexual abuse. Adolescents from nuclear families had 90% higher odds of being in a higher abuse category compared to those from extended families. Finally, the level of parental education was a significant factor for sexual abuse. Using university-level education as the reference, adolescents whose parents had only a primary-level education had 113% higher odds of being in a higher category of sexual abuse.

**Table 5: Predictors of Sexual Abuse**

| Predictors | OR | 95% CI | Wald (p-value) |
| --- | --- | --- | --- |
| Age of Mother | 0.97 | (0.94, 1.01) | 2.25 (.13) |
| Residential Area (Ref: Urban) |  |  |  |
| Rural | 0.70 | (0.37, 1.31) | 1.26 (.26) |
| Semi-urban | 0.40 | (0.21, 0.77) | 7.50 (.01) |
| Gender (Ref: Female) |  |  |  |
| Male | 0.27 | (0.17, 0.44) | 28.02 (<.001) |
| Family Structure (Ref: Extended Family) |  |  |  |
| Single Parenthood | 2.34 | (0.96, 5.70) | 3.53 (.06) |
| Nuclear Family | 1.90 | (1.20, 3.00) | 7.46 (.01) |
| Parent physical illness (Ref: Yes) |  |  |  |
| No | 0.63 | (0.38, 1.06) | 3.08 (.07) |
| Parent mental illness (Ref: Yes) |  |  |  |
| No | 1.86 | (1.00, 3.46) | 3.84 (.05) |
| Parental education (Ref: University level) |  |  |  |
| Illiterate | 1.63 | (0.44, 5.99) | 0.54 (.46) |
| Primary level | 2.13 | (1.09, 4.18) | 4.83 (.028) |
| Secondary level | 1.15 | (0.68, 1.95) | 0.27 (.60) |
| Income (Ref: Upper class) |  |  |  |
| Lower class | 0.79 | (0.43, 1.45) | 0.58 (.45) |
| Middle Class | 1.05 | (0.60, 1.83) | 0.03 (.86) |
| Ethnicity (Ref: Brahmin/Chhetri) |  |  |  |
| Dalits | 1.51 | (0.70, 3.26) | 1.10 (.29) |
| Indigenous/Minorities group | 0.86 | (0.53, 1.41) | 0.34 (.56) |

**Notes:** ***OR*** *= Odds Ratio;* ***CI*** *= Confidence Interval (Lower, Upper). The Nagelkerke R-squared value indicated modest explanatory power (.141). The degrees of freedom (df) for each Wald test = 1. There was no significant evidence to suggest a violation of the proportional odds assumption, supporting the appropriateness of using ordinal regression for the analysis.*

**Predictors of child neglect**

Logistic regression analysis identified significant risk factors for neglect (Table 6). Adolescents residing in semi-urban areas had 65% lower odds of experiencing higher levels of neglect compared to adolescents in urban areas. Adolescents from the Indigenous/Minorities group had 1.72 times higher odds of experiencing neglect compared to those from Brahmin/Chhetri group. The odds of experiencing higher neglect severity were 43% lower for male adolescent compared to female adolescent.

**Table 6. Predictors of child neglect.**

| Variable | OR | 95% CI | Wald (p-value) |
| --- | --- | --- | --- |
| Age of mother | 1.00 | (0.97, 1.03) | 0.03 (0.87) |
| Residential Area (Ref: Urban) |  |  |  |
| Rural | 0.99 | (0.59, 1.65) | 0.00 (0.97) |
| Semi-urban | 0.35 | (0.20, 0.60) | 14.20 (< 0.001) |
| Gender (Ref: Female) |  |  |  |
| Male | 0.57 | (0.40, 0.82) | 9.06 (0.002) |
| Family structure (Ref: Extended family) |  |  |  |
| Single Parenthood | 1.40 | (0.66, 2.98) | 0.77 (0.38) |
| Nuclear | 1.14 | (0.79, 1.66) | 0.51 (0.48) |
| Parent physical illness |  |  |  |
| Yes | 0.87 | (0.58, 1.30) | 0.50 (0.48) |
| Parent mental illness |  |  |  |
| Yes | 1.29 | (0.76, 2.20) | 0.89 (0.35) |
| Parental education (Ref: University) |  |  |  |
| Illiterate | 1.67 | (0.57, 4.89) | 0.86 (0.36) |
| Primary level | 1.06 | (0.58, 1.94) | 0.04 (0.84) |
| Secondary level | 0.98 | (0.64, 1.50) | 0.01 (0.92) |
| Income (Ref: Upper class) |  |  |  |
| Lower class | 0.99 | (0.61, 1.62) | 0.01 (0.97) |
| Middle class | 0.81 | (0.51, 1.29) | 0.80 (0.37) |
| Ethnicity (Ref: Brahmin/Chhetri group) |  |  |  |
| Dalits | 1.58 | (0.78, 3.18) | 1.62 (0.20) |
| Indigenous/Minorities group | 1.72 | (1.17, 2.52) | 7.58 (0.006) |

**Note: *OR*** *= Odds Ratio;* ***CI*** *= Confidence Interval (Lower, Upper). Nagelkerke R² = .087).*

**Predictors of Domestic violence**

Family structure was a significant predictor of domestic violence. Using extended families as the reference group, adolescents from single-parent families had 184% higher odds of being in a higher category of domestic violence. Parental education was also a significant predictor. Using university-level education as the reference group, adolescents from illiterate families had 193% higher odds of being in a higher category of domestic violence. Furthermore, adolescent ethnic background was associated with the outcome. When using the Brahmin/Chhetri group as the reference group, adolescents from the Indigenous/Minorities group had 63% higher odds of being in a higher category of domestic violence.

**Table 7: Predictors of Domestic Violence**

| Predictor | OR | 95% CI | Wald (p-value) |
| --- | --- | --- | --- |
| Age of mother | 0.98 | (0.95, 1.01) | 2.52 (.11) |
| Residential Area (ref: Urban) |  |  |  |
| Rural | 0.77 | (0.49, 1.22) | 1.22 (.27) |
| Semi-urban | 0.73 | (0.49, 1.08) | 2.48 (.12) |
| Gender (ref: Female) |  |  |  |
| Male | 0.74 | (0.55, 1.00) | 3.67 (.06) |
| Family structure (ref: extended) |  |  |  |
| Single parenthood | 2.84 | (1.50, 5.37) | 10.31 (.001) |
| Nuclear Family | 1.27 | (0.93, 1.74) | 2.26 (.13) |
| Parent medical illness (ref: Yes) |  |  |  |
| No | 1.20 | (0.86, 1.68) | 1.12 (.29) |
| Parent mental illness (ref: Yes) |  |  |  |
| No | 1.51 | (0.96, 2.37) | 3.22 (.07) |
| Parental education (ref: University level) |  |  |  |
| Illiterate | 2.93 | (1.15, 7.49) | 5.05 (.03) |
| Primary level | 1.10 | (0.65, 1.85) | 0.12 (.73) |
| Secondary level | 1.01 | (0.70, 1.45) | 0.00 (.96) |
| Income (ref: Upper class) |  |  |  |
| Lower class | 0.88 | (0.58, 1.34) | 0.35 (.56) |
| Middle Class | 0.78 | (0.53, 1.16) | 1.54 (.21) |
| Ethnicity (Ref: Brahmin/Chhetri group) |  |  |  |
| Dalits | 1.38 | (0.75, 2.55) | 1.08 (.30) |
| Indigenous/Minorities group | 1.63 | (1.17, 2.27) | 8.38 (.004) |

***Notes: OR*** *= Odds Ratio;* ***CI*** *= Confidence Interval (Lower, Upper). The Nagelkerke R-squared value indicated modest explanatory power (.067). The degrees of freedom (df) for each Wald test = 1. There was no significant evidence to suggest a violation of the proportional odds assumption, supporting the appropriateness of using ordinal regression for the analysis.*

**Discussion**

This study identified multiple socio-demographic and familial risk factors associated with various forms of violence exposure in a clinical sample of adolescents in Nepal.

*Caste/Ethnicity*

Adolescent psychiatric patients from the Dalit group (socially marginalized Hindu caste) had 86% higher odds of being in a higher physical abuse category compared to patients from the Brahmin/Chhetri group (dominant Hindu castes). Adolescent patients from the Indigenous/Minorities group faced 72% higher odds of neglect and 63% higher odds of being in a higher category of domestic violence compared to the same dominant castes group. These findings align with prior research from Nepal in the general population documenting a disproportionate burden of child maltreatment among socially marginalized groups [23,35]. Longstanding structural inequalities such as caste-based discrimination, limited access to education, healthcare, and poverty may increase household stress leading to increased violence [22,23]. In Nepal, socially marginalized communities like Dalits and indigenous groups encounter barriers for accessing child protection services. These include structural obstacles like geographic remoteness and lack of education, as well as social and cultural challenges such as stigma, institutional mistrust, and a lack of culturally and linguistically appropriate services [10,21,22,36].

*Gender*

Gendered vulnerabilities were most apparent in the context of sexual abuse and neglect, with girls experiencing significantly higher risks among adolescent patients. The odds for males were 73% lower for the category of sexual abuse than the odds for females, indicating that girls were disproportionately targeted, aligning with robust global evidence [37,38] and even supported by review study done in Asia [39]. Moreover, the 75% greater odds of neglect for girls suggest prevailing gender-based discrimination in caregiving and resource allocation, especially in traditional societies like Nepal where sons are often prioritized [10-12,40]. Moreover, deep-rooted patriarchal norms and male-dominated social structures had historically privileged men, granting them disproportionate power and perpetuating systemic exploitation and marginalization of women [41].

*Place of residence*

The adolescent patients residing in urban areas who comprised 64% of the sample, had significantly higher odds of experiencing all three categories of abuse category, as well as an increased risk of neglect. These findings align with patterns observed in general population studies from countries such as the United States [42], Nepal [43], and other LMICs [37]. In many LMICs, including Nepal, rapid urbanization has led to overcrowded living conditions, unplanned settlements, and overburdened public infrastructure. Consequently, adolescents in urban areas may face heightened risks of emotional and sexual violence due to reduced community surveillance, overcrowding, and the increased anonymity of urban life [24-26]. Additionally, in such environments, families may experience heightened economic pressures, which can contribute to elevated levels of parental stress and reduce emotional availability for children [44]. The social dislocation and cultural stress associated with internal migration even when motivated by seeking better opportunities may constitute a risk factor for increased violence [45].

It should be noted that participants in the present study were adolescent patients from diverse regions and residential settings across the country. However, adolescents from urban areas were more, due to relatively easier access to the hospital, whereas those from rural and semi-urban areas were comparatively underrepresented due to logistical and access-related challenges.

*Mother’s Age*

The present study found that each additional year in mother's age corresponded to a 3% decrease in the odds of being in a higher emotional abuse category and a 4% decrease of being in a higher physical abuse category. Young maternal age is a well-documented risk factor for heightened child violence, a finding consistent across both global and local contexts [13-15]. According to other studies, the practice of child marriage remains entrenched in Nepal despite legal prohibitions against it, affecting 33% of girls before the age of 18 and 8% of girls before the age of 15 [46,47]. This prematurely ends formal education for young girls, limiting their future economic opportunities and trapping them into a cycle of poverty known as a catalyst for family stress and violence [16,17]. Beyond economic strain, younger mothers often face developmental challenges that exacerbate parenting stress. They typically have less parenting experience, greater difficulties with emotional regulation, and have limited access to social support networks. This combination of factors can increase frustration and reduce coping mechanisms, leading to an increased reliance on abusive disciplinary strategies [46]. Young motherhood is not confined to low-income countries; even in high-income nations like Canada and Australia, younger maternal age is linked to a greater reported use of physical punishment [48,49].

*Family structure*

Adolescent patients from single-parent families had 98% higher odds of being in a higher category of emotional abuse and 90% higher odds of being classified in a more severe category of sexual abuse compared to adolescents from extended families. Additionally, the odds of being in a higher category of domestic violence were 184% higher for adolescents from single-parent relatives to their counterparts from extended families. These results echo well-established findings from international studies [50-52], emphasizing the protective role of extended family networks [13,14]. The extended family including grandparents, aunts, uncles, and other relatives serves as a vital protective buffer against violence towards children. By offering consistent supervision, emotional support, and shared caregiving responsibilities, it may play a crucial role in shielding adolescents from abuse and neglect [53,54].

In the context of Nepal, the extended family structure is particularly vital for supporting families when parents migrate for work abroad. Labor migration is widespread in Nepal, with many parents compelled to work abroad to meet their families’ basic needs. This often results in prolonged family separation, disrupting traditional child-rearing practices and weakening the emotional and practical support systems within the household [55]. The absence of one or both parents can create a care vacuum, reduce supervision and increase the risk of neglect and violence for children left behind [37, 56]. These family-level challenges are further compounded by broader systemic issues, including pervasive poverty and an under-resourced child welfare system that lacks the capacity to provide effective oversight and support [57].

*Parental education level*

Adolescents whose parents had only a primary-level education had 113% higher odds of experiencing more severe sexual abuse. Similarly, the odds of being in a higher category of domestic violence were 193% higher among those from illiterate families. Our findings align with previous studies indicating that lower levels of parental education are strongly associated with increased risks of child neglect in resource-deprived settings [18-20]. The present study contributes to the growing evidence that low parental education is a risk factor for child violence, likely by limiting awareness of children's rights and increasing vulnerability to stress. This underscores the urgent need to break the intergenerational cycle of disadvantage in Nepal through grassroots initiatives that promote education and child rights awareness.

**Strengths and Limitations**

*Strengths*: There are several strengths of the present study. First, the chosen population ensures sufficient variability in the violence variables, allowing for robust analysis of association with socio-demographic and familial risk factors. Second, the study also benefits from a comprehensive assessment of multiple forms of abuse, supported by rigorous quantitative analysis. Third, importantly, it provides policy-relevant insights into high-risk groups, offering valuable guidance for targeted interventions. While the findings align with global research, they also contribute new, context-specific knowledge relevant to Nepal. Overall, the results highlight the importance of considering socio-demographic and familial risk factors when addressing violence exposure among adolescent psychiatric patients and in planning interventions accordingly.

*Limitations:* The main limitation of the present study is the generalizability of its findings, as the results from a clinical sample of adolescent psychiatric patients. It is essential to note that the relatively small and specific sample, drawn from the only Nepali psychiatric hospital unit, may have introduced selection bias, thereby limiting the generalizability of the results. As a result, the observed patterns of violence may not fully reflect the differences in experiences of adolescents from rural or marginalized communities.

Second, cross-sectional design restricts the ability to infer causality. Although associations between violence exposure and socio-demographic or familial factors were identified, the directionality or temporal sequence of these relationships cannot be determined. To prevent the stigmatization of minorities and Indigenous peoples, it is especially important to highlight positive aspects of the group, particularly when they do not have significantly higher rates of violence and abuse, and even have a lower rate compared to the majority.

Third, the reliance on self-reported, quantitative measures introduces the possibility of recall bias and underreporting, especially given the sensitive nature of topics such as abuse and neglect. Cultural stigma, fear, or discomfort in disclosing such experiences may have led some participants to withhold information, potentially leading to conservative estimates of violence prevalence. Moreover, measuring violence solely by frequency overlooks critical qualitative aspects such as severity and context, potentially simplifying a complex, multifaceted phenomenon. Another limitation is that some important factors related to the individuals, their families, and the adolescents’ surroundings were not measured in our study.

**Future recommendations**

Despite its limitations, this study offers valuable preliminary evidence to guide future research and inform the development of culturally appropriate interventions. Based on our findings, we recommend more in-depth and nuanced research studies to better understand the complex interplay of multiple risk factors contributing to violence among adolescent psychiatric patients in the Nepali context. Future studies should explore the cultural dimensions of violence, particularly the role of caste hierarchies and rigid gender norms. These deeply embedded societal structures not only heighten adolescents’ risk of experiencing violence but also serve as significant barriers to disclosure and access to care. A more thorough investigation into these cultural dynamics could yield critical insights and help shape interventions that are both effective and culturally sensitive. Thus, it is essential to develop targeted interventions, including community-based parenting programs for young and single parents and economic empowerment modules integrated with mental health services. To improve access, training Female Community Health Volunteers (FCHVs) and school nurses or school counselor in violence identification and establishing school-based referral systems.

Additionally, the impact of urban violence on adolescents’ exposure to abuse demands further attention. Urban settings may present unique challenges such as overcrowding, poverty, and social fragmentation that interact with individual and familial vulnerabilities. This area of inquiry would benefit from a comprehensive approach that considers environmental, social, and psychological factors.

Importantly, future research should adopt an intersectional framework to examine how overlapping vulnerabilities such as caste, gender, and urban or rural residence, compound the risk of violence. Mixed methods approach combining quantitative data with qualitative insights could capture the complexity of these experiences more effectively and develop culturally adapt measurement tools.

Finally, policymakers should prioritize interventions that address systemic inequalities, including caste-based discrimination and urban stressors, while also expanding mental health services to underserved rural and marginalized communities. Developing culturally sensitive, equity-focused policies will be essential for protecting vulnerable adolescents and promoting long-term mental health outcomes.

**Conclusion**

This study sheds light on the socio-demographic and familial risk factors associated with violence exposure among adolescents receiving psychiatric care in Nepal. The findings underscore an urgent need for targeted interventions aimed at protecting this particularly vulnerable population. Specific risk factors such as socially marginalized caste and ethnic backgrounds, urban stressors, gender disparities, young motherhood, single-parent households, and low parental education must be addressed through culturally sensitive and contextually informed strategies.

Policymakers and mental health practitioners should incorporate these insights into the design of preventive measures and therapeutic programs tailored to the needs of at-risk adolescents. Such efforts are essential for reducing violence exposure and its long-term psychological consequences.

Finally, there is a clear need for further research in this field. Future studies should adopt longitudinal and mixed methods approaches to better capture the mechanisms, trajectories, and cultural dimensions of violence exposure in the Nepali context. This will be crucial for developing sustainable, evidence-based interventions that effectively support the mental health and well-being of vulnerable youth.

**Acknowledgement**

We are deeply grateful to all the participants and their parents for their time, cooperation, and willingness to contribute. We also wish to express our sincere gratitude to the CWIN-Nepal and the Child and Adolescent Psychiatry Unit at Kanti Children’s Hospital for their generous support and collaboration, which played a crucial role in the successful completion of this research.

**References**

1. Hillis S, Mercy J, Amobi A, Kress H. Global prevalence of past-year violence against children: a systematic review and minimum estimates. *Pediatrics*. 2016;137(3)\:e20154079.
2. Haller AC, Zoellner F, Klasen F, Petermann F, Barkmann C, Otto C, et al. Long-term consequences of externalizing behavior problems. *Kindh* *Entwickl*. 2016 Jan;25(1):31-40. doi: 10.1026/0942-5403/a000186.
3. Schlack R, Peerenboom N, Neuperdt L, Junker S, Beyer AK. The effects of mental health problems in childhood and adolescence in young adults: Results of the KiGGS cohort. *J Health Monit*. 2021;6(4):3-19.
4. Copeland WE, Wolke D, Shanahan L, et al. Adult functional outcomes of common childhood psychiatric problems: a prospective, longitudinal study. *JAMA* *Psychiatry*. 2015;72(9):892–899.
5. Tzouvara V, Kupdere P, Wilson K, Matthews L, Simpson A, Foye U. Adverse childhood experiences, mental health, and social functioning: A scoping review. *Child Abuse Negl.* 2023;139:106092.
6. Colizzi M, Lasalvia A, Ruggeri M. Prevention and early intervention in youth mental health. *Int J Ment Health Syst*. 2020;14:23.
7. Alloh FT, Regmi P, Onche I, Teijlingen E, Trenoweth S. Mental Health in LMICs: Going beyond the need for funding. *Health Prospect*. 2018;17(1):12–17.
8. Palermo T, Pereira A, Neijhoft N, Bello G, Buluma R, Diem P, et al. Risk factors for childhood violence and polyvictimization: a cross-country analysis from three regions. *Child Abuse Negl*. 2019;88:348–61.
9. Fry D, McChesney S, Padilla K, Ivantury S. Violence against children in South Asia: a systematic review of evidence since 2015 [Internet]. Kathmandu: UNICEF; 2020 [cited 2024 Sep 18]. 148 p. Available from: https://www.unicef.org/rosa/media/9916/file/Full%20Report.pdf
10. United Nations Population Fund Nepal. Literature review on harmful practices in Nepal [Internet]. Kathmandu (NP): *UNFPA Nepal*; 2017. <https://nepal.unfpa.org/sites/default/files/pub-pdf/Literature%20Review%20on%20Harmful%20Practices%20in%20Nepal.pdf>.
11. Perrin N, Marsh M, Clough A, et al. Social norms and beliefs about gender-based violence scale. *Confl Health*. 2019;13:6.
12. Rechenberg T, Fleischer T, Sander C, et al. Gender-related stigma and childhood violence. *BMC Public Health*. 2024;24(1):2396.
13. Schneider W. Single mothers, the role of fathers, and the risk for child maltreatment. *Child Youth Serv Rev.* 2017;81:81-93.
14. Lopes AI, Leal J, Sani AI. Parental Mental Health Problems and Risk of Child Maltreatment. *Societies*. 2021;11(3):108.
15. Kisely S, Strathearn L, Najman JM. Risk Factors for Maltreatment in Siblings of Abused Children. *Pediatrics*. 2021;147(5):e2020036004.
16. Beatriz E, Salhi C. Child discipline and socioeconomic disparities in LMICs. *Child Abuse Negl*. 2019;94:104023.
17. Maguire-Jack K, Sattler K. Neighborhood poverty and child maltreatment. *J Interpers Violence*. 2023;38(5-6):4814-4831.
18. Chaulagain A, Kunwar A, Watts S, et al. Child and adolescent mental health problems in Nepal. *Int J Ment Health Syst*. 2019;13:53.
19. Pesando LM, De Cao E, La Mattina G, et al. Status inconsistency and violent parenting. *Soc Sci Med.* 2024;351:116954.
20. Maguire-Jack K. Community context of child maltreatment. *J Aggress Maltreat Trauma*. 2014;23(3):229–248.
21. Mishra S. The unjust treatment of Dalits in Nepal [Internet]. Genocide Watch Blog. 2024 May. Available from: https://www.genocidewatchblog.com/post/the-unjust-treatment-of-dalits-in-nepal
22. UNFPA Nepal. Literature review on harmful practices in Nepal. [https://nepal.unfpa.org/sites/default/files/pub- pdf/Literature%20Review%20on%20Harmful%20Practices%20in%20Nepal.pdf](<https://nepal.unfpa.org/sites/default/files/pub-pdf/Literature%20Review%20on%20Harmful%20Practices%20in%20Nepal.pdf>)
23. Pariyar B, Lovett JC. Dalit identity in urban Pokhara, Nepal. *Geoforum*. 2016;75:134-47. doi:10.1016/j.geoforum.2016.07.006.
24. Ochnik D, Buława B, Nagel P, et al. Urbanization and mental health: A network analysis. *Sci Rep*. 2024;14:24974.
25. Akins N. Stress and the City: The impacts of city living and mental health. *Scripps Senior Theses*. 2022;1892.
26. Chant S, Klett-Davies M, Ramalho J. Challenges for adolescent girls in urban settings. *GAGE*. 2017 Jul. [https://www.gage.odi.org/wp-content/uploads/2019/01/Young-Female-Adolescents-in-Urban-Areas-RER-FINAL.pdf](https://www.gage.odi.org/wp-content/uploads/2019/01/Young-Female-Adolescents-in-Urban-Areas-RER-FINAL.pdf)
27. Rai Y, Gurung D, Gautam K. Insight and challenges: mental health services in Nepal. *BJPsych Int*. 2021;18(2)\:E5.
28. Government of Nepal, Office of the Prime Minister and Council of Ministers. National Population and Housing Census 2021 (National Report). *National Statistics Office, Kathmandu, Nepal*. March 2023; 1:84.
29. Countrymeters. Nepal population (Live) [Internet]. Countrymeters. Available from: https://countrymeters.info/en/Nepal
30. Shakil A, Day PG, Chu J, Woods SB, Bridges K. PedHITSS: A screening tool to detect childhood abuse in clinical settings. *Fam Med*. 2018;50(10):763–769. <https://doi.org/10.22454/FamMed.2018.778329>
31. Zolotor AJ, Runyan DK, Dunne MP, Jain D, Péturs HR, Ramirez C, Volkova E, Deb S, Lidchi V, Muhammad T, Isaeva O. ISPCAN Child Abuse Screening Tool Children's Version (ICAST-C): Instrument development and multi-national pilot testing. *Child Abuse Negl*. 2009 Nov;33(11):833-41. doi:10.1016/j.chiabu.2009.09.004. PMID:19857897
32. International Society for the Prevention of Child Abuse and Neglect (ISPCAN). ICAST abuse screening tools [Internet]. Available from: <https://www.ispcan.org/learn/icast-abuse-screening-tools/?v=402f03a963ba>
33. Joshi SK, Acharya K. Modification of Kuppuswamy’s socioeconomic status scale in the context of Nepal. *Kathmandu Univ Med J (KUMJ)*. 2019 Jan-Mar;17(65):1-2. PubMed PMID: 31734669.
34. Sah R, Brøndbo PH, Ma J, Hansen KL, Devkota N, Handegård BH, Javo AC. Prevalence of violence in a clinical sample of adolescent patients visiting a child and adolescent psychiatric outpatient clinic in Nepal. *PLOS ONE*, 2025; 20(10): e0335396. https://doi.org/10.1371/journal.pone.0335396
35. Kandel P, Kunwar R, Karki S, Kandel D, Lamichhane P. Child maltreatment in Nepal: Prevalence and associated factors. Public Health. 2017;151:106–13. doi:10.1016/j.puhe.2017.06.020
36. Alink LR, Euser S, van IJzendoorn MH, Bakermans-Kranenburg MJ. Is elevated risk of child maltreatment in immigrant families associated with socioeconomic status? Evidence from three sources. Int J Psychol. 2013;48:117–27. doi:10.1080/00207594.2012.734622
37. Cerna-Turoff I, Fang Z, Meierkord A, Wu Z, Yanguela J, Bangirana CA, Meinck F. Factors Associated With Violence Against Children in Low- and Middle-Income Countries: A Systematic Review and Meta-Regression of Nationally Representative Data. *Trauma Violence Abuse*. 2021 Apr;22(2):219-232. doi: 10.1177/1524838020985532. Epub 2021 Jan 19. PMID: 33461441; PMCID: PMC7961628.
38. Stoltenborgh, M., Bakermans-Kranenburg, M. J., Alink, L. R., & van IJzendoorn, M. H. The prevalence of child maltreatment across the globe: Review of a series of meta-analyses. *Child Abuse Review*. (2014); 24, 37-50. doi:10.1002/car.2353
39. Moody G, Cannings-John R, Hood K, Kemp A, Robling M. Establishing the international prevalence of self-reported child maltreatment: a systematic review by maltreatment type and gender. *BMC Public Health*. 2018;18(1):1164. doi:10.1186/s12889-018-6044-y.
40. Ghimire, A., & Samuels, F. (2020). *A tale of contradictions: Understanding the impact of social norms on Nepali men and boys.* Policy Brief, Gender and Adolescence: Global Evidence. London, UK.
41. Dahal P, Joshi SK, Swahnberg K. A qualitative study on gender inequality and gender-based violence in Nepal. *BMC Public Health*. 2022 Nov 1;22(1):2005. doi:10.1186/s12889-022-14389-x
42. Beatriz ED, Salhi C, Griffith JL, Molnar BE. Urbanicity matters in self-reported child maltreatment prevalence: Findings from a nationally representative study. *Child Abuse Negl*. 2018;79:371-83. doi:10.1016/j.chiabu.2018.02.028.
43. Atteraya, MS, Ebrahim, NB, & Gnawali, S.. Determinants of child maltreatment in Nepal: Results from the 2014 Nepal Multiple Indicator Cluster Survey (the 2014 NMICS). *Child Abuse & Neglect*. 2018;76: 400–407.
44. Ren X, Singh S, Bhutoria A, Doğan H. Placemaking through time in Nepal: conceptualising the historic urban-rural landscape of Kathmandu. *Urban Plan* [Internet]. 2025. Available from: https://doi.org/10.17645/up.8947
45. Singh RP, Dhakal J. Problems and Prospects of Urbanization in Kathmandu Valley. *Int J Atharva*. 2024;2(1):19-33. doi:10.3126/ija.v2i1.62821
46. Seta R. Child marriage and its impact on health: a study of perceptions and attitudes in Nepal. *J Glob Health Rep*. 2023;7:e2023073. doi:10.29392/001c.88951
47. Girls Not Brides. Child marriage atlas: Nepal [Internet]. Girls Not Brides. Available from: <https://www.girlsnotbrides.org/learning-resources/child-marriage-atlas/atlas/nepal/>
48. King B, Fallon B, Goulden A, O’Connor C, & Filippelli, J. What Constitutes Risk of Future Maltreatment Among Young Mothers? An Examination of Child Protection Investigations in Ontario, Canada. *Families in Society*. 2019;100(4), 409-421. https://doi.org/10.1177/1044389419847319 (Original work published 2019)
49. Kisely S, Strathearn L, Najman JM. Risk factors for maltreatment in siblings of abused children. *Pediatrics*. 2023;151(2):e2022059283. doi:10.1542/peds.2022-059283
50. Euser S, Alink LR, Pannebakker F, Vogels T, Bakermans-Kranenburg MJ, van IJzendoorn MH. The prevalence of child maltreatment in the Netherlands across a 5-year period. *Child Abuse Negl*. 2013; 37:841–51. doi: 10.1016/j.chiabu. 2013.07.004
51. Tran NK, van Berkel SR, van IJzendoorn MH, Alink LRA. Child and family factors associated with child maltreatment in Vietnam. *J Interpers Violence*. 2018;36(5–6):NP2931–NP2953. doi:10.1177/0886260518767914
52. Stith SM, Liu T, Davies LC, Boykin EL, Alder MC, Harris JM, Dees JEMEG. Risk factors in child maltreatment: A meta-analytic review of the literature. *Aggress Violent Behav*. 2009;14:13–29. doi:10.1016/j.avb.2006.03.006
53. Sigdel A, Shah S, & Kumar, A. Self-reported child abuse in the home: a cross-sectional survey of prevalence, perpetrator characteristics and correlates among public secondary school students in Kathmandu, Nepal. *BMJ Open*. 2018.
54. Lama DB, Pun A, Maharjan R. The evolution of alternative care in Nepal over the last decade and way forward. *South Asian J Soc Stud Econ*. 2023. doi:10.1177/23493003231187708
55. Simkhada PP, Regmi PR, Van Teijlingen E, Aryal N. Identifying the gaps in Nepalese migrant workers’ health and well-being: a review of the literature. *J Travel Med*. 2017 Jul 1;24(4)\:tax021. doi:10.1093/jtm/tax021.
56. Adhikari DBP. The consequences of mother international migration to the left behind girls under 16 for their education, health & psycho-social development in Chitwan District. *OCEM J Manag Technol Soc Sci*. 2019;1(1):7-20. Available from: https://journal.oxfordcollege.edu.np/index.php/ojmts/article/view/3
57. UNICEF Nepal. (2021). For Every Child, Protection. <https://www.unicef.org/nepal/reports/every-child-protection>
