# Supplementary material for: Violence exposure among adolescent psychiatric patients in Nepal: Socio-demographic and familial risk factors
Source: PLoS One. 2026 Aug 14;21(8):e0355947. doi: 10.1371/journal.pone.0355947 (PMC13475971; doi:10.1371/journal.pone.0355947)
Supplement: Supplementary file 3 [file pone.0355947.s005.docx]

**Manuscript ID: PONE-D-25-60069**

**Title- Violence Exposure Among Adolescent Psychiatric Patients in Nepal: Socio-Demographic and Familial Risk Factors**

**Response to the reviewers**

Thank you for your letter dated 29^th^ April 2026 and for the thoughtful and constructive comments regarding our manuscript entitled *Violence Exposure Among Adolescent Psychiatric Patients in Nepal: Socio-Demographic and Familial Risk Factors.* Your feedback has helped us significantly to improve the quality of our paper. We have carefully addressed all comments raised by both the Academic Editor and Reviewer 2, as requested. Below is our point‑by‑point response to each concern raised. All changes have been highlighted / tracked in the revised manuscript file submitted herewith. We believe the manuscript is now substantially strengthened and we hope it meets the journal's standards for publication. Thank you again for your time and guidance.

**The requirements made by the Academic Editor**

**ABSTRACT:**

**Requirement 1:** The abstract is excessively lengthy and should be limited to approximately 300 words. It is recommended to condense the content while retaining key findings and essential information.

**Our response**: We apologize for the lengthy abstract and have followed your advice to condense the content while retaining key findings and essential information. The abstract is now shortened and consists of 292 words. The revised version of the whole abstract has been added to the manuscript (lines 21-57).

**Requirement 2:** In the abstract, please specify the age range and characteristics of the adolescent participants along with the clinical setting.

**Our response**: Thank you for this reminder. We have now added the following information in the Methods part of the abstract: *A total of 810 adolescents aged 11–15 years (392 boys, 418 girls) at Kanti Children’s Hospital in Kathmandu, were included.* (lines 30-31).

**Requirement 3**: Clarify who provided which data.

**Our response**: We have clarified who provided which data in the Methods part: *Adolescents reported past-year exposure to violence using adapted PedHITSS and ICAST-C items, while parents provided socio-demographic and family data.* (lines 31-33).

**Requirement 4**: Briefly describe the questionnaire used, whether standardized or adapted tools, statistical software and analytical approach

**Our response:** Thank you for your suggestion. We have added the following, informing about the instruments used, analytical approach, and statistical software: “*Adolescents reported past-year violence exposure using a modified version of PedHITSS and ICAST-C items, while parents provided socio-demographic and family data. Associations were examined using ordinal and logistic regression in SPSS 29*.” (lines 31-34).

**Requirement 5**: Instead of stating “several socio-demographic and familiar factors” please explicitly mention the key variables that showed significant associations.

**Our response:** Thank you for pointing this out. We have now explicitly mentioned the key variables that showed significant associations, see the revised Results part of the abstract (lines 36-51).

**Requirement 6:** Provide appropriate statistical estimates (odds ratios, confidence intervals, p- values) for the findings.

**Our response:** As requested, we have added the appropriate statistical estimates for all findings throughout the revised Results part of the abstract (lines 36-51).

**Requirement 7:** The phrase “higher odds” appears frequently, you may consider using varied expressions such as “increased likelihood” or “greater risk”

**Our response:** We have now varied expressions according to your advice, using expressions like: “was more frequent” and “linked to increased…”, see the revised Results part of the abstract (lines 36-51)

**Requirement 8:** Consider streamlining the presentation for better readability

**Our response:** We have streamlined the presentation of the abstract for better readability – see the totally revised abstract version (line 21-56).

**Requirement 9:** Begin the Conclusion part with a clear summary of key findings based on your objective followed with concise implications.

**Our response:** Thank you for your suggestion. We have now revised the Conclusion part by providing a clear summary of key findings, followed by implications of our findings (lines 52-57).

**BACKGROUND:**

**Requirement 10:** Please incorporate regarding the perception of “child abuse” and “neglect” in Nepal context as well. The contextual scenario has been missed in justification of the research.

**Our response:** Thank you for this important comment. In response, we have added a paragraph to the Introduction that focuses on context and highlights cultural / contextual influences on the perception of child abuse and neglect, with particular emphasis on the Nepali context (lines 73-90).

**Requirement 11:** The statement “Gaining deeper understanding of this population is particularly urgent” needs more clarification. What aspect has been intended to explain here?

**Our response:** Thank you for your comment. We have reformulated the sentence and clarified the meaning as follows: “*Such knowledge is particularly important in a context where mental health resources are scarce, and early intervention systems remain underdeveloped*” (lines 114-116).

**METHODS**

**Requirement 11:** Participants: Justify the recruitment of this age group (11-15 years). As the study was conducted in CAP-OPD, the information regarding “patients were admitted to the hospital irrespective of their caste or ethnic background” is confusing.

**Our response:** We appreciate your comment and agree that our choice of age group requires clarification. Child and adolescent psychiatric outpatient services typically serve individuals from early childhood up to 16–18 years, with some variation across settings. However, due to hospital regulations at the study site (Kanti Children’s Hospital), children above 15 years are not admitted. We therefore included the oldest eligible group (11–15 years), although the broader age group (11–18 years) would have been preferable. As our data on maltreatment were based on self-reports, we focused on adolescents, as individuals aged 11–15 years are generally considered to have the cognitive and linguistic maturity to reliably complete self-administered screening instruments. We have added this information in the revised manuscript (lines 131-134).

To avoid confusion, we have moved the information about the hospital admitting children from all ethnic groups to the previous paragraph (“Study Site and Population”) (lines 123-126). In the following paragraph (“Participants”), we specify that participants were categorized based on caste and ethnicity for the purposes of this study (lines 143-152).

**Requirement 12:** Sample size calculation: How did you calculate the sample size and mention its adequacy.

**Our response:** The adequacy of the sample size will vary for different research questions depending on what the smallest meaningful effect size(s) would be, the measurement level of the dependent variable, and to some extent the amount of information and variation in both outcome variables and predictors. Unfortunately, we did not consider all power analytic aspects, but rather collected an as large as possible sample on the basis of a one-year time-frame We have added the following sentence in the Participants section: “*The sample size was primarily determined by the fixed one-year data collection period at the clinic, during which 810 eligible participants were enrolled consecutively.”* (lines 140-142).

**Requirement 13:** Did you take written consent from the child as well?

**Our response:** These sentences have been added at the onset of the Procedure section of the manuscript: *“Before data collection, all participating adolescents and parents were informed about the project, both orally and in written form. Written, informed consent was obtained from all participants, i.e., both adolescents and their parents*” (lines 155-157).

**Requirement 14:** How long did it take to complete the questionnaire?

**Our response:** Filling in the instruments about maltreatment took approximately 20-30 minutes. However, adolescents were not given a time limit to complete them. The following sentence has now been added to the manuscript: “*Completing the maltreatment questionnaires took approximately 20–30 minutes. However, adolescents were not given a fixed time limit to complete them*.” (lines 162-164)

**Requirement 15:** Which method of data collection was used? Any unexpected occurring aroused or not? Such as emotional break up?

**Our response:** Thank you for advising us to add this information. The following sentences have been added to the manuscript: “*Data was collected using a self-administered, paper-based questionnaire completed by adolescents, while socio-demographic and family data were provided by one parent (either mother or father) and recorded manually by staff. Data collection was carried out by two office secretaries and one research assistant, all of whom received training, supervision, and monitoring from the first author throughout the data collection period. Completing the maltreatment questionnaires took approximately 20–30 minutes. However, adolescents were not given a fixed time limit to complete them. No unexpected incidents (e.g., emotional distress) were observed during the procedure”* (lines 157-165).

**Requirement 16:** How the researchers assured the validity of the collected data as both parents were there even if collected differently.

**Our response:** Thank you for your comments. We agree that additional information is needed to clarify this. We have revised the manuscript, adding the following paragraph to clarify the procedure: *To assure the validity of the sensitive data provided by the adolescents, the forms were completed by each adolescent alone in a separate, quiet room, prior to a clinical consultation, and with trained secretaries available to answer any queries and to clarify possible misun­derstandings. Subsequently, the clinicians responsible reviewed the filled-in forms that the participants brought with them to the consultation room. Data plotting was done manually throughout the collection period*” (lines 165-171).

**Requirement 17:** How were the instruments translated? Which translation process was used?

**Our response:** Thank you for this reminder. We have now made the information more precise. The following sentences have been included at the end of the Instruments section: “*All instruments were translated into Nepali and then back translated by bilingual clinicians and professional translators to ensure accuracy and cultural relevance. The translation of tools was done with the standard procedure suggested by the WHO.*” (line 205 - 207).

**Requirement 18:** Validity and reliability of the questionnaire?

**Our response:** Thank you for your suggestion. We agree that this information should be included. The following text on the validity and reliability of the PedHITSS questionnaire has been added to the manuscript (indicated here in bold): “*This 5-item questionnaire was originally designed to detect and prompt provider investigation into child abuse in clinical settings and has been validated against the Conflict Tactics Scale: Parent-Child Version (CTSPC)* ***demonstrating good internal consistency (α = .85) and strong concurrent validity (rₛ = .70, p < .01)*** (lines 182-183)

**Requirement 19:** It seems that there occurred clinical and legal issues requiring attention. Please elaborate more that because of this research participants did not get hurt and no harm was done.

**Our response:** Thank you for highlighting this important matter. Additional sentences have been added to elaborate more: “*The study adhered to the ethical principle of non-maleficence, ensuring that no harm was done to any participant. Clinicians responsible for each patient reviewed all completed forms. Any cases of abuse identified through the screening process that required clinical intervention or legal action were promptly managed by the attending clinicians in accordance with established institutional protocols, and in coordination with the principal researcher. Thus, adolescent who needed future help were referred to the One Stop Crisis Management Center (OCMC) at Kanti Children’s Hospital and, where deemed appropriate, to the Child Workers in Nepal Concerned Centre (CWIN-Nepal) helpline, protection services and other needed services. This approach ensured participant safety while maintaining the integrity of the research procedures.*” (lines 230-239)

**Requirement 20:** For those participants who frequently experienced sexual abuse, physical abuse, neglect what was the researchers’ role on it?

**Our response:** The researcher's role was to conduct the assessments about child abuse and neglect. Next, the patients moved on to meet with the clinicians responsible, taking with them the filled-in forms for the clinician to see. Identified participants with higher frequency of child maltreatment were referred by the clinicians to the One Stop Crisis Management Center (OCMC) at Kanti Children’s Hospital and, where deemed appropriate, to the Child Workers in Nepal Concerned Centre (CWIN-Nepal) helpline, protection services and other needed services.

**DISCUSSION**

**Requirement 21:** Do not just include the higher odds. Discuss your findings with meaningful interpretation highlighting the contextual scenario.

**Our response:** Thank you for this advice. We have reformulated the information about the results in the Discussion section (for Caste/Ethnicity, lines 412-413; for Gender, lines 427-433; for Family Structure, lines 474-477; and for Parental Education, lines 494-498). Contextual scenario is provided for all findings. Finally, we have added a few lines about the consequences of limited access to child protection services for marginalized groups to clarify meanings (lines 423-425),

**Requirement 22:** How do you explain the limited sample size?

**Our response:** Thank you for raising your concern. The following sentence about the limited sample size has been added in the revised manuscript under “Participants”:

*The sample size was primarily determined by the fixed one-year data collection period at the clinic, during which 810 eligible participants were enrolled consecutively*. (140-142).

In the “Limitations” of the Discussion, we have further elaborated:

*Also, the relatively small and specific sample may have introduced selection bias, limiting the extent to which the findings can be generalized to the broader clinical adolescent population. Access to outpatient mental health services in Nepal is influenced by factors such as family socioeconomic status, parental education, and mental health awareness. Adolescents from families with greater resources or higher self-efficacy may be more likely to seek and obtain care, meaning that the present sample may not fully represent all adolescents with mental health problems in the clinical population.* (lines 520-530)

Further, in response to your question, we would like to add that when we considered using power analysis to estimate sample size, we thought that a power analysis that just focuses on one of the research questions, or a strategy to collect data only within a relatively short time-frame were not optimal to secure enough power for each research question in the study.

**Requirement 23:** Also, the setting of this study reflects the tertiary referral hospital thus, consider these as well when writing limitation.

**Our response:** In response to your suggestion, we have included the following text in the “Limitations” of the Discussion section: “*We acknowledge that the findings from a hospital-based child and adolescent psychiatric OPD may not be fully generalizable to non-clinical populations or to adolescents in other settings or regions*” (lines 521-523).

**CONCLUSIONS**

**Requirement 24:** Mention clearly what are the key findings of this study first as of the objective. In the current writing, it is not known how the findings underscore an urgent need for targeted interventions.

**Our response:** Thank you for your comment. We have revised the Conclusions section to present the key findings and conclusions at the outset, in line with the study objectives and results. We have also removed the statement indicating an urgent need for targeted interventions. The revised Conclusions now read as follows:

“*This study demonstrated that violence exposure among adolescent psychiatric patients in Nepal is associated with a range of socio-demographic and familial factors. Elevated risks were observed among adolescents from socially marginalized castes, indigenous and minority groups, and urban areas, as well as among females. Additional risk factors included younger maternal age, single-parent households, and parental illiteracy. These patterns appear to be embedded within and partly shaped by the broader social, cultural, and structural context of Nepal.*

*The findings highlight the importance of systematically addressing socio-demographic and familial risk factors in the assessment and treatment of adolescents with a history of violence exposure. Moreover, they underscore the need to develop culturally informed prevention and intervention strategies tailored to at-risk adolescent populations”* (lines 554-566).

**The requirements made by Reviewer 2**

**Requirement 1:** The manuscript is excessively lengthy and would benefit from substantial reduction. The authors are encouraged to improve conciseness by eliminating redundant content and enhancing overall readability.

**Our response**: Thank you for your valuable suggestion. We have carefully revised the manuscript to improve the language and to increase conciseness, clarity, and readability throughout. Earlier the total words count was 8251 whereas at present it had been reduced to 7920 words count. We believe the revised manuscript is now more concise and easier to follow.

**Requirement 2:** The conclusions are not adequately articulated. The authors should revise the conclusions in both the abstract and the main conclusion section to ensure they accurately reflect the key findings of the study

**Our response**: Thank you for your comment. The conclusions have been revised to ensure that they reflect key findings in the Abstract’s Conclusions section (lines 52-57).

**ABSTRACT**

**Requirement 3:** Line 21: The abstract is excessively lengthy and should be limited to approximately 300 words. It is recommended to condense the content while retaining key findings and essential information.

**Our response:** The Abstract section has now been properly shortened in accordance with the requirement while retaining key findings and essential information with word count of 292 (lines 23-57).

**Requirement 4:** Line 44: Basic sociodemographic characteristics of the participants, including sample size, age distribution, and sex, should be clearly presented to provide readers with an immediate overview of the study population.

**Our response:** Thank you for pointing this out. The following information has now been added: “*A total of 810 adolescents aged 11–15 years (392 boys, 418 girls) at Kanti Children’s Hospital in Kathmandu, were included.*” (lines 30-31)

**Requirement 5:** Line 46: Terms such as “higher” are vague and do not convey precise information; therefore, exact numerical values should be included throughout the manuscript, including in the sections discussed below.

**Our response:** We agree that more precise information should be given and have now included exact numerical values both in the Abstract section, Results part (lines 36-51) and throughout the manuscript.

**Requirement 6:** Line 55: Similarly, protective factor should also be presented with corresponding numerical data to enhance clarity and interpretability.

**Our response:** Thank you. We have provided numerical data for rural / semi-urban residency and higher maternal age (i.e., protective factors) as well to ensure consistency with the presentation of risk factors. (lines 40-47)

**Requirement 7:** Line 58: This is not necessary. It has been mentioned in objective of the study already. Instead, the abstract should emphasize the major findings and key conclusions of your study.

**Our response:** Thank you for your suggestion. We have revised the “Conclusions” of the Abstract to better summarize the main findings and highlight the key conclusions. It now reads as follows: “ *Child* *maltreatment was more frequent among Dalit and Indigenous/minority groups, females, and urban residents, and was associated with younger maternal age, single-parent households, and parental illiteracy. These findings underscore the need for culturally informed, trauma-focused prevention and intervention within adolescent mental health services*”. (lines 53-57).

.
**INTRODUCTION**

**Requirement 8**: Line 75: To strengthen the rationale, it is advisable to include comparative data on the prevalence of violence in both low- and middle-income countries (LMICs) and high-income countries (HICs). This will better justify the need for the present study.

**Our response:** Thank you for your suggestion. Accordingly, we have added the following paragraph to better justify the need for the present study: “*According to WHO estimates based on retrospective reports from adults, the global lifetime prevalence of child maltreatment is 23–28% for physical abuse, around 36% for emotional abuse, and around 18% for sexual abuse. [1]. However, these global averages may obscure substantial disparities between low- and middle-income countries (LMICs) and high-income countries (HICs). In Nepal, this burden is markedly elevated, with 81–83% of children subjected to violent discipline and nearly 50% experiencing physical maltreatment [7,8]. In LMICs such as Nepal, the convergence of entrenched socio-cultural norms, poverty, and social inequality amplifies adolescents’ exposure to multiple forms of violence and poor mental health [9]. Despite increasing global attention, research on the socio-demographic and familial factors that predispose adolescents to violence exposure remains limited, including those in LMICs [10,11]. Studies focusing on clinical populations are even more scarce.* (lines 73-85)

**Requirement 9**: Line 83: Several paragraphs, particularly in the introduction, are overly long and could be merged and streamlined for improved readability. Sentences should be shortened and made more concise.

**Our response:** Thank you for your suggestion. We have revised the Introduction section to improve readability and clarity while adding some new information as suggested by the reviewers.

**MATERIALS AND METHODS**

**Requirement 10**: Line 113: Begin with the sentence mentioning study site at first to provide immediate contextual clarity.

**Our response:** Thank you for your suggestion. The Materials and Methods section has been reorganized to start with Study Design, followed by Study Site and Population. The latter section has been substantially shortened and now provides context more concisely, as follows: “*The study was conducted at the Child and Adolescent Psychiatry Outpatient Unit (CAP-OPD) of Kanti Children’s Hospital, a tertiary referral center in Kathmandu, Nepal. The hospital serves a diverse patient population from all geographic regions, castes and ethnic groups, and from different socioeconomic backgrounds. The study population comprised children and adolescents attending the CAP-OPD during the study period (15 January 2023 to 15 January 2024)*”. (lines 123-129)

**Requirement 11:** Line 122: Some statements require proper citation to support their validity. Please ensure that all claims are evidence-based and appropriately referenced.

**Our response:** The more detailed and surplus information about the CAP-OPD requiring proper citation, has been removed to shorten the text.

**Requirement 12**: Line 127: Add “years old”

**Our response:** Thank you. We have added “years”. It now reads: “*Eligible participants were adolescents aged 11–15 years …”* (line 132)

**Requirement 13**: Line 182: The instruments section is overly detailed and should be summarized into a single concise paragraph highlighting only the most relevant aspects.

**Our response:** Thank you for your suggestion. We have revised the entire “Instruments” section to improve conciseness and have shortened the part about the screening instruments of child maltreatment to highlight only the most relevant aspects (lines 178-207).

**RESULTS**

**Requirement 14:** Line 272: Clarification is needed regarding parental education. It is unclear whether data were collected for one parent or both. If both were included, clearly explain how differences were handled. This should be explicitly described in the methodology section.

**Our response:** Thank you for the comment. We have added the following information to the “Background Information Questionnaire” paragraph: “*Data on parental education were collected from both parents. In households with two parents, the higher level of education was used.*” (lines 211-214). In the Results section, we revised the text as follows: “*Most households had secondary-level education as its maximum (48.5%), followed by households with at least one university-educated parent (36.6%)*”. (lines 271-273).

**Requirement 15:** Line 326: Odds ratios (ORs) better to be reported directly, not converted into percentages. It is not the preferred scientific reporting style for regression results. Report odds ratio with confidence interval and p-value. Address this issue throughout the paper.

**Our response:** Thank you for the suggestion. We have presented the OR, CI and P -values both in the tables and text throughout the paper as recommended.

**Requirement 16:** Line 369: Similarly, clarification is required for variables related to parental mental illness. Specify whether this was assessed for one or both parents, how it was measured, and how it was classified in the study.

**Our response:** Thank you for this clarification request. To clarify, we have added the following sentence to the “Background Questionnaire” section: “*Data on parents’ physical and mental health histories were collected for both parents in dichotomous (yes/no) form. If either parent had a chronic condition, the variable was coded as “yes.”* (lines 214-217).

**DISCUSSION**

**Requirement 17:** Line 410: Detailed numerical data should generally not be presented in the Discussion section, as this may duplicate information already reported in the Results. Instead, the Discussion should focus on interpreting the key findings and highlighting their implications.

**Our response:** Thank you for your suggestion. We have removed all detailed numerical data from the discussion section to avoid duplicate information and have focused on interpreting the key findings and highlighting their implications.

**Requirement 18:** Line 498: Is sexual abuse classified in your study? How did you mention it as a severe?
**Our response:**

All types of abuse, including sexual abuse, were categorized as “Never,” “Rarely,” “Fairly often,” and “Frequently,” as described in the “Instruments” section. The term “severe” used in the Discussion referred to the “Frequently” category; however, we agree that alternative terminology should be avoided. The text has now been revised to: “*Adolescents from families with low or no parental education showed higher risks of more frequent sexual abuse and domestic violence.*” (lines 493-496)

**CONCLUSION**

**Requirement 19:** Line 580: This paragraph appears to reflect recommendations rather than the conclusions of the study. It is recommended to revise this section to clearly present the key conclusions drawn from the study findings, ensuring alignment with the stated objectives and results.

**Our response:** Thank you for your suggestions. We have revised the Conclusions section to more clearly present the key conclusions drawn from the study findings, ensuring alignment with the stated objectives and results (lines 554-566).
