# Supplementary material for: Violence exposure among adolescent psychiatric patients in Nepal: Socio-demographic and familial risk factors
Source: PLoS One. 2026 Aug 14;21(8):e0355947. doi: 10.1371/journal.pone.0355947 (PMC13475971; doi:10.1371/journal.pone.0355947)
Supplement: Supplementary file 4 [file pone.0355947.s006.docx]

**Manuscript ID: PONE-D-25-60069**

**Title- Violence Exposure Among Adolescent Psychiatric Patients in Nepal: Socio-Demographic and Familial Risk Factors**

**Response to the reviewers**

Thank you for your letter dated 26^th^ June 2026 and for the thoughtful and constructive comments regarding our manuscript entitled *Violence Exposure Among Adolescent Psychiatric Patients in Nepal: Socio-Demographic and Familial Risk Factors.* Your feedback has helped us to improve the quality of our paper and we have carefully addressed all comments raised by the Academic Editor, as requested. Below is our point‑by‑point response to each concern raised. All changes have been highlighted / tracked in the revised manuscript file submitted herewith.

In addition to the revisions requested by the reviewers, we identified minor inaccuracies in the frequencies and percentages reported in Table 2 and in the corresponding text. These have been corrected. To improve consistency with the analytical approach, we also revised the table heading to: *“Distribution of violence exposure outcome variables included in the regression analyses (n = 714).”* (lines 314-341) for Table 2 and “*Distribution of socio-demographic and family-related predictor variables included in the regression analyses (n=714)” for Table 1(*lines 287-290, 304-305*).*

We hope that these additional revisions can be accepted by the journal and that all revisions made to the manuscript adequately address the remaining concerns. We believe the manuscript is now substantially strengthened and we hope it meets the journal's standards for publication. Thank you again for your time and guidance.

**The requirements made by the Academic Editor**

Abstract

**Requirement 1:**

Background:

The background section is concise; however, it would benefit from additional context regarding the importance of violence exposure and the existing gaps in literature. Consider including a brief statement that more clearly aligns with the study title and strengthens the rationale for the research. In addition, the term “maltreatment” may not fully encompass the broader concept of “violence exposure.” Using “violence exposure” consistently throughout the abstract and manuscript would better reflect the study title, methodology, and overall focus of the research.

**Our response:** Thank you for this feedback which strengthens the rationale of the study and clarifies the concept used for this study (child violence). We have now revised the background part of the abstract, providing additional context by addressing both the importance of the topic and existing gaps in literature. The text now reads:

*“Violence exposure is a major public health concern among adolescents. Yet, evidence on socio-demographic and familial risk factors in adolescent psychiatric populations in Nepal and other low- and middle-income countries is limited”.* (lines 23- 26)

Further, we have now changed the word “maltreatment” to “violence exposure” in accordance with your suggestion, both in the abstract (line 54) and also throughout the manuscript.

**Requirement 2:**

Introduction:

The authors have provided relevant contextual information; however, similar to the concern raised for the abstract, terminology requires greater clarity. The background should first introduce the broader concept of violence exposure and then discuss specific forms such as abuse and maltreatment. Currently, the text appears to equate violence exposure with maltreatment, which may lead to conceptual ambiguity, as maltreatment represents only one aspect of violence exposure. Therefore, it is recommended that the authors introduce violence exposure as the overarching construct and subsequently position maltreatment within this broader framework, rather than beginning the discussion directly with maltreatment.

**Our response:** Thank you for noting that the terminology that we use requires greater clarity, we quite agree that this is needed. We have now changed the overarching concept that we use, from “maltreatment” to “violence exposure”. Further, we have added a sentence early in the introduction of the manuscript to define the concept of child violence which includes maltreatment as one of its components. The sentence runs as follows:

*“Violence exposure encompasses a broad range of experiences, including child maltreatment (physical, emotional, and sexual abuse and neglect), witnessing domestic or community violence, peer victimization and bullying, and other forms of interpersonal violence [1]” (*lines-69-72*)*

**Requirement 3:**

Participants: Please specify when the data collection period was.?: Line 132-133 “Eligible participants were adolescents aged 11–15 years who attended the unit during this period” is not clear.

**Our response:** Thank you for pointing this out. The following sentence has now been added for clarification:

“*The study population comprised adolescents aged 11-15 years during the data collection period 15 January 2023 to 15 January 2024.”* (lines 136-137)

**Requirement 4:**

It is suggested to rewrite this sentence “As data on maltreatment were obtained through self-reports, only adolescents were selected as informants, given their cognitive and linguistic maturity to reliably complete self-administered screening instruments.” to make it more academic writing.

**Our response:** Thank you for your comment. We have added the following paragraph to make it more academic writing: ***“****Only adolescents were selected as informants, given their cognitive and linguistic maturity to reliably complete self-administered screening instruments. Adolescents with diagnoses such as intellectual developmental disorder (IDD), acute psychosis, or any other condition that could compromise their cognitive and linguistic capacity to reliably complete self-administered maltreatment screening instruments, were excluded”* (lines 137-142)

**Requirement 5:**

Sample size:

Thank you for the clarification provided in the response letter. However, this explanation should also be incorporated into the manuscript to ensure reader's understanding. In addition, the concern regarding sample size adequacy has not been fully resolved. Please provide a clear justification, as outlined in the response letter, including the number of outcome events and the number of predictors included in the models. This information is essential for readers to appropriately assess the robustness and validity of the study findings.

**Our response:** We thank you for highlighting the need for a clearer explanation of sample size adequacy. We have now made the needed explanation for the readers within the Statistical Analysis section of the manuscript under the subheading: “*Sample adequacy*”*.* The new text reads as follows:

“*The sample size was primarily determined by the fixed one-year data collection period at the clinic, during which 810 eligible participants were enrolled consecutively. Of the 810 participants enrolled in the study, 31 were excluded due to missing data, and an additional 65 participants were excluded from the predictor analyses due to their classification in the "other" ethnicity category, which comprised a heterogeneous group. This exclusion reduced model complexity and allowed for a more clearly defined ethnicity variable. Consequently, the predictor analyses were conducted on a final analytical sample of 714 participants”.* (lines 275-283).

We have also revised the manuscript to explicitly report the number of outcome events and the number of predictors included in the ordinal logistic regression models. We added the following paragraph:

*“Our regression models included nine predictor variables (15 estimated parameters, excluding threshold parameters). Events-per-parameter (EPP) criterion for sample size adequacy states that approximately 10 events per estimated parameter is the conventional recommendation [36]. EPP values below this threshold may reduce the precision and stability of odds ratio estimates and increase the risk of overfitting [37]. The present study had the smallest number of cumulative events involved in the ordinal regression contained 83 cases for physical abuse, 32 for sexual abuse, 148 for emotional abuse, 173 for neglect and 66 for domestic violence, corresponding to EPP values ranging from approximately 2.1 to 11.5.”* (lines 265-274)

**Requirement 6:**

Limitation section:

The information mentioned in the limitation section regarding a relatively small sample does not justify this study’s limitation. Please consider your sample size with the number of your predictors so that the sample size will not be a limitation.

**Our response:** Thank you for your kind suggestion to revise the limitation section of the Discussion chapter. We acknowledge that several models did not achieve the conventional EPP benchmark of 10 recommended by Peduzzi et al. Consequently, we cannot claim that the sample size was fully adequate for all regression analyses, particularly for outcomes with relatively few severe cases, such as sexual abuse and domestic violence. To ensure transparency, we have now explicitly acknowledged this limitation in the manuscript and noted that these findings should be interpreted with appropriate caution because limited EPP may reduce the precision and stability of the estimated associations and increase the risk of overfitting, as discussed by Peduzzi et al. (1996) and Babyak (2004).

The following information has now been added to the limitation section to explicitly detail our strategy to ensure model stability and robustness, and including also information on EPP ratios:

*Moreover, several ordinal logistic regression models had EPP ratios below the conventional recommendation of 10, which may have reduced the precision and stability of odds ratio estimates and increased overfitting risk. Accordingly, these findings should be interpreted with caution.”* (lines 548-551)

**Requirement 7:**

Line 155-157: Written informed consent from both adolescents and parents, or ascent from adolescents? Please check

**Our response:** We are sorry for mixing up. Now it has been corrected as follows:

“*Written assent was obtained from all adolescents, and written informed consent was obtained from their parents, legal guardians or caregivers*.” (lines 163-164).

**Requirement 8:**

Line 205-207: “All instruments were translated into Nepali and then back-translated by bilingual clinicians and professional translators to ensure accuracy and cultural relevance. The translation of tools was done with the standard procedure suggested by the WHO”. Citation required.

**Our response:** Thank you. Now we have added reference no 34 (line 215).
