# Supplementary material for: Violence exposure among adolescent psychiatric patients in Nepal: Socio-demographic and familial risk factors
Source: PLoS One. 2026 Aug 14;21(8):e0355947. doi: 10.1371/journal.pone.0355947 (PMC13475971; doi:10.1371/journal.pone.0355947)
Supplement: Peer review history

# Peer review: Decision Letter 0

**DOI:** 10.1371/journal.pone.0355947.r001

**Authors:** Bimala Panthee

28 Apr 2026

PLOS One

Dear Dr. Sah,

Thank you for submitting your manuscript to PLOS ONE. After careful consideration, we feel that it has merit but does not fully meet PLOS ONE’s publication criteria as it currently stands. Therefore, we invite you to submit a revised version of the manuscript that addresses the following points raised during the review process.

====================================

Abstract:

Methods:

- Please specify the age range and characteristics of the adolescent participants along with the clinical setting.
- Clarify who provided which data
- Briefly describe the questionnaire used whether standardized or adapted tools, statistical software and analytical approach

Results:

The results are interesting but could be presented more clearly

- Instead of stating “several socio-demographic and familiar factors” please explicitly mention the key variables that showed significant associations.
- Provide appropriate statistical estimates (odds ratios, confidence intervals, p values) for the findings.
- The phrase “higher odds” appears frequently, you may consider using varied expressions such as “increased likelihood” or “greater risk”
- Consider streamlining the presentation for better readability

Conclusion:

- Begin with a clear summary of key findings based on your objective followed with concise implications.

Background:

Please incorporate regarding the perception of “child abuse” and “neglect” in Nepal context as well. The contextual scenario has been missed in justification of the research. The statement “Gaining deeper understanding of this population is particularly urgent” needs more clarification. What aspect has been intended to explain here?

Methods

Participants: Justify the recruitment of this age group (11-15 years). As the study was conducted in CAP-OPD, the information regarding “patients were admitted to the hospital irrespective of their caste or ethnic background” is confusing.

Sample size calculation: How did you calculate the sample size and mention it’s adequacy

Procedure

Did you take written consent from the child as well?

How long did it take to complete the questionnaire? Which method of data collection was used? Any unexpected occurring aroused or not? Such as emotional break up? How the researchers assured the validity of the collected data as both parents were there even if collected differently.

Instruments

How the instruments were translated? Which translation process was used?

Validity and reliability of the questionnaire?

Ethical consideration

It seems that there occurred clinical and legal issues requiring attention. Please elaborate more that because of this research participants did not get hurt and no harm was done.

Results

For those participants who frequently experienced sexual abuse, physical abuse, neglect what was the researchers’ role on it?

Discussion

Do not just include the higher odds. Discuss your findings with meaningful interpretation highlighting the contextual scenario.

Limitation

How do you explain the limited sample size?

Also, the setting of this study reflects the tertiary referral hospital thus, consider these as well when writing limitation.

Conclusion

Mention clearly what are the key findings of this study first as of the objective. In the current writing, it is not known how the findings underscore an urgent need for targeted interventions.

====================================

Please submit your revised manuscript by Jun 12 2026 11:59PM. If you will need more time than this to complete your revisions, please reply to this message or contact the journal office at plosone@plos.org. When you're ready to submit your revision, log on to https://www.editorialmanager.com/pone/ and select the 'Submissions Needing Revision' folder to locate your manuscript file.

Please include the following items when submitting your revised manuscript:

- A letter that responds to each point raised by the academic editor and reviewer(s). You should upload this letter as a separate file labeled 'Response to Reviewers'.
- A marked-up copy of your manuscript that highlights changes made to the original version. You should upload this as a separate file labeled 'Revised Manuscript with Track Changes'.
- An unmarked version of your revised paper without tracked changes. You should upload this as a separate file labeled 'Manuscript'.

If you would like to make changes to your financial disclosure, please include your updated statement in your cover letter. Guidelines for resubmitting your figure files are available below the reviewer comments at the end of this letter.

If applicable, we recommend that you deposit your laboratory protocols in protocols.io to enhance the reproducibility of your results. Protocols.io assigns your protocol its own identifier (DOI) so that it can be cited independently in the future. For instructions see: https://journals.plos.org/plosone/s/submission-guidelines#loc-laboratory-protocols. Additionally, PLOS ONE offers an option for publishing peer-reviewed Lab Protocol articles, which describe protocols hosted on protocols.io. Read more information on sharing protocols at https://plos.org/protocols?utm_medium=editorial-email&utm_source=authorletters&utm_campaign=protocols.

As the corresponding author, your ORCID iD is verified in the submission system and will appear in the published article. PLOS supports the use of ORCID, and we encourage all coauthors to register for an ORCID iD and use it as well. Please encourage your coauthors to verify their ORCID iD within the submission system before final acceptance, as unverified ORCID iDs will not appear in the published article. *Only*  the individual author can complete the verification step; PLOS staff *cannot*  verify ORCID iDs on behalf of authors.

We look forward to receiving your revised manuscript.

Kind regards,

Bimala Panthee

Academic Editor

PLOS One

Journal Requirements:

When submitting your revision, we need you to address these additional requirements.

1. Please ensure that your manuscript meets PLOS ONE's style requirements, including those for file naming. The PLOS ONE style templates can be found at

https://journals.plos.org/plosone/s/file?id=wjVg/PLOSOne_formatting_sample_main_body.pdf and https://journals.plos.org/plosone/s/file?id=ba62/PLOSOne_formatting_sample_title_authors_affiliations.pdf

2. Please provide a complete Data Availability Statement in the submission form, ensuring you include all necessary access information or a reason for why you are unable to make your data freely accessible. If your research concerns only data provided within your submission, please write "All data are in the manuscript and/or supporting information files" as your Data Availability Statement.

3. We note that the grant information you provided in the ‘Funding Information’ and ‘Financial Disclosure’ sections do not match.

When you resubmit, please ensure that you provide the correct grant numbers for the awards you received for your study in the ‘Funding Information’ section.

4. Please update your submission to use the PLOS LaTeX template. The template and more information on our requirements for LaTeX submissions can be found at http://journals.plos.org/plosone/s/latex.

5. We note you have included a table to which you do not refer in the text of your manuscript. Please ensure that you refer to Table 4, 5, 7 in your text; if accepted, production will need this reference to link the reader to the Table.

6. Please include captions for your Supporting Information files at the end of your manuscript, and update any in-text citations to match accordingly. Please see our Supporting Information guidelines for more information: http://journals.plos.org/plosone/s/supporting-information.

7. We are unable to open your Supporting Information file [Paper II data analysis Plos One.sav]. Please kindly revise as necessary and re-upload.

8. We note that there is identifying data in the Supporting Information file < PLOS ONE Global inclusivity.docx, PONE-D-25-20724 paper.pdf>. Due to the inclusion of these potentially identifying data, we have removed this file from your file inventory. Prior to sharing human research participant data, authors should consult with an ethics committee to ensure data are shared in accordance with participant consent and all applicable local laws.

Data sharing should never compromise participant privacy. It is therefore not appropriate to publicly share personally identifiable data on human research participants. The following are examples of data that should not be shared:

-Name, initials, physical address

-Ages more specific than whole numbers

-Internet protocol (IP) address

-Specific dates (birth dates, death dates, examination dates, etc.)

-Contact information such as phone number or email address

-Location data

-ID numbers that seem specific (long numbers, include initials, titled “Hospital ID”) rather than random (small numbers in numerical order)

Data that are not directly identifying may also be inappropriate to share, as in combination they can become identifying. For example, data collected from a small group of participants, vulnerable populations, or private groups should not be shared if they involve indirect identifiers (such as sex, ethnicity, location, etc.) that may risk the identification of study participants.

Additional guidance on preparing raw data for publication can be found in our Data Policy (https://journals.plos.org/plosone/s/data-availability#loc-human-research-participant-data-and-other-sensitive-data) and in the following article: http://www.bmj.com/content/340/bmj.c181.long.

Please remove or anonymize all personal information (<specific identifying information in file to be removed>), ensure that the data shared are in accordance with participant consent, and re-upload a fully anonymized data set. Please note that spreadsheet columns with personal information must be removed and not hidden as all hidden columns will appear in the published file.

9. If the reviewer comments include a recommendation to cite specific previously published works, please review and evaluate these publications to determine whether they are relevant and should be cited. There is no requirement to cite these works unless the editor has indicated otherwise.

Additional Editor Comments :

Upon internal evaluation of the reviews provided, we kindly request you to disregard the reviewer report provided by Reviewer 1. No amendments are required in response to Reviewer 1’s comments.

[Note: HTML markup is below. Please do not edit.]

Reviewers' comments:

Reviewer's Responses to Questions

**Comments to the Author**

1. Is the manuscript technically sound, and do the data support the conclusions?

Reviewer #1: Partly

Reviewer #2: Partly

**********

2. Has the statistical analysis been performed appropriately and rigorously?

Reviewer #1: Yes

Reviewer #2: Yes

**********

3. Have the authors made all data underlying the findings in their manuscript fully available?

The PLOS Data policy

Reviewer #1: Yes

Reviewer #2: Yes

**********

4. Is the manuscript presented in an intelligible fashion and written in standard English?

Reviewer #1: Yes

Reviewer #2: Yes

**********

Reviewer #1: yes i want add additional comment in different portion.Title: Violence Exposure Among Adolescent Psychiatric Patients in Nepal: Socio-Demographic and Familial Risk Factors

Short Title: Risk Factors for violence in Nepali Adolescent Psychiatric Youth

Keywords: Violence exposure, Nepal, risk factors, adolescent, clinical sample

Abstract

Background

Exposure to violence during adolescence is a major public health issue, especially for those already experiencing mental health challenges. Adolescents receiving psychiatric care often report higher levels of violence exposure compared to their peers. While global studies have identified various socio-demographic and family-related risk factors linked to such exposure, there is a significant research gap in low- and middle-income countries like Nepal—particularly among clinically referred adolescents.

Objectives

This study aimed to identify socio-demographic and familial risk factors associated with different forms of violence exposure among adolescents receiving care at a hospital-based child and adolescent psychiatric unit in Nepal.

Methods

A cross-sectional study design was conducted using, with data collected through structured questionnaires administered to caregivers to obtain socio-demographic and familial information, and to adolescents to assess past-year exposure to violence. Statistical analysis was performed using IBM SPSS Statistics, with ordinal and logistic regression models applied to examine the associations between different forms of violence exposure and potential risk factors, including gender, caste/ethnicity, residential setting, family structure, parental education, maternal age, and parental medical history.

Results

The analysis revealed several socio-demographic and familial factors significantly associated with adolescents’ exposure to violence. Adolescents from the Dalit caste were more likely to experience physical abuse compared to their Brahmin/Chhetri peers. Those from Indigenous/Minority (Janajati) groups had increased odds of both neglect and domestic violence.

Female adolescents were at higher risk for emotional abuse, sexual abuse, and neglect. Urban residency was linked to greater likelihood of emotional abuse and neglect.

Family structure influenced risk patterns: adolescents from single-parent households were more likely to experience emotional abuse and domestic violence, whereas those from nuclear families faced higher odds of sexual abuse compared to adolescents from extended families. Lower parental education was associated with increased sexual abuse, and parental illiteracy correlated with higher domestic violence.

Conversely, older maternal age appeared protective, reducing the likelihood of both physical and emotional abuse.

Conclusions

This study highlights that adolescents attending psychiatric services in Nepal are differentially exposed to various forms of violence based on their socio-demographic and familial characteristics. The findings emphasize the critical importance of integrating considerations of intersecting vulnerabilities—such as female gender, marginalized caste/ethnic background, urban residence, younger maternal age, and lower parental education—into the design of preventive programs and tailored clinical interventions aimed at mitigating violence exposure in this high-risk population.

Key Word: arrange in alphabetic orders

Order of Authors: Add task disturbation.

Financial Disclosure: at least type of find and who is provided fund need to mention. add funding details.

Ethics Statement: The study was approved by the Kanti Children's Hospital Ethical Review Committee

(Reference No: 621) and the Nepal Health Research Council (Reference No: 1227).

Introduction: adequate information to support the clarify the study

Method : Arrange the method part

1. Research design: This study used a cross-sectional, quantitative research design

2. Setting: give the complete information about research setting

3. Population: clearly mention about research population.

4. Sample Size: How you chooses the sample, and what was the sample distribution.

5. Sampling Technique: clearly mention sampling technique

6. Data collection Procedure: duration of data collection,

7. Statistical Analysis: mention clearly Differential and inferential statistics

8. Result: Socio-demographic data- arrange in order wise variable, Table description mention just below the table.

9. Discussion part: write discussion based on objectives. Like socio-demographic information discussion write under socio-demographic heading, and so on.

10. Conclusion: Before limitation and recommendation write conclusion. Conclusion should be based on research findings. Policy making portion you will recommended.

11. Limitation : Mention point wise

12. Recommendation:

Reviewer #2: Title: Violence Exposure Among Adolescent Psychiatric Patients in Nepal: Socio-Demographic and Familial Risk Factors

The manuscript is generally well written; however, several revisions are required to improve its clarity and scientific rigor. Specific comments have been provided within the manuscript for the authors’ consideration.

In general;

1. The manuscript is excessively lengthy and would benefit from substantial reduction. The authors are encouraged to improve conciseness by eliminating redundant content and enhancing overall readability.

2. The conclusions are not adequately articulated. The authors should revise the conclusions in both the abstract and the main conclusion section to ensure they accurately reflect the key findings of the study

In detail:

Abstract

• Line 21: The abstract is excessively lengthy and should be limited to approximately 300 words. It is recommended to condense the content while retaining key findings and essential information.

• Line 44: Basic sociodemographic characteristics of the participants, including sample size, age distribution, and sex, should be clearly presented to provide readers with an immediate overview of the study population.

• Line 46: Terms such as “higher” are vague and do not convey precise information; therefore, exact numerical values should be included throughout the manuscript, including in the sections discussed below.

• Line 55: Similarly, protective factor should also be presented with corresponding numerical data to enhance clarity and interpretability.

• Line 58: This is not necessary. It has been mentioned in objective of the study already. Instead, the abstract should emphasize the major findings nd key conclusions of your study.

Introduction

• Line 75: To strengthen the rationale, it is advisable to include comparative data on the prevalence of violence in both low- and middle-income countries (LMICs) and high-income countries (HICs). This will better justify the need for the present study.

• Line 83: Several paragraphs, particularly in the introduction, are overly long and could be merged and streamlined for improved readability. Sentences should be shortened and made more concise.

Methods and Materials

• Line 113: Begin with the sentence mentioning study site at first to provide immediate contextual clarity.

• Line 122: Some statements require proper citation to support their validity. Please ensure that all claims are evidence-based and appropriately referenced.

• Line 127: Add “years old”

• Line 182: The instruments section is overly detailed and should be summarized into a single concise paragraph highlighting only the most relevant aspects.

Results

• Line 272: Clarification is needed regarding parental education. It is unclear whether data were collected for one parent or both. If both were included, clearly explain how differences were handled. This should be explicitly described in the methodology section.

• Line 326: Odds ratios (ORs) better to be reported directly, not converted into percentages. It is not the preferred scientific reporting style for regression results. Report odds ratio with confidence interval and p-value. Address this issue throughout the paper.

• Line 369: Similarly, clarification is required for variables related to parental mental illness. Specify whether this was assessed for one or both parents, how it was measured, and how it was classified in the study.

Discussion

• Line 410: Detailed numerical data should generally not be presented in the Discussion section, as this may duplicate information already reported in the Results. Instead, the Discussion should focus on interpreting the key findings and highlighting their implications.

• Line 498: Is sexual abuse classified in your study? How did you mention it as a severe?

Conclusion

• Line 580: This paragraph appears to reflect recommendations rather than the conclusions of the study. It is recommended to revise this section to clearly present the key conclusions drawn from the study findings, ensuring alignment with the stated objectives and results.

**********

what does this mean?). If published, this will include your full peer review and any attached files.

If you choose “no”, your identity will remain anonymous but your review may still be made public.

**Do you want your identity to be public for this peer review?**  For information about this choice, including consent withdrawal, please see our Privacy Policy

Reviewer #1: **Yes:** Janaki Dhami

Reviewer #2: No

**********

[NOTE: If reviewer comments were submitted as an attachment file, they will be attached to this email and accessible via the submission site. Please log into your account, locate the manuscript record, and check for the action link "View Attachments". If this link does not appear, there are no attachment files.]

To ensure your figures meet our technical requirements, please review our figure guidelines: https://journals.plos.org/plosone/s/figures

You may also use PLOS’s free figure tool, NAAS, to help you prepare publication quality figures: https://journals.plos.org/plosone/s/figures#loc-tools-for-figure-preparation.

NAAS will assess whether your figures meet our technical requirements by comparing each figure against our figure specifications.

Attachment Submitted filename: PLOS One.docx

Attachment Submitted filename: paper II- final PLOS ONE_SS.docx

# Author comment: Author response to Decision Letter 1

**DOI:** 10.1371/journal.pone.0355947.r002

8 Jun 2026

Manuscript ID: PONE-D-25-60069

Title- Violence Exposure Among Adolescent Psychiatric Patients in Nepal: Socio-Demographic and Familial Risk Factors

Response to the reviewers

Thank you for your letter dated 29th April 2026 and for the thoughtful and constructive comments regarding our manuscript entitled Violence Exposure Among Adolescent Psychiatric Patients in Nepal: Socio-Demographic and Familial Risk Factors. Your feedback has helped us significantly to improve the quality of our paper. We have carefully addressed all comments raised by both the Academic Editor and Reviewer 2, as requested. Below is our point by point response to each concern raised. All changes have been highlighted / tracked in the revised manuscript file submitted herewith. We believe the manuscript is now substantially strengthened and we hope it meets the journal's standards for publication. Thank you again for your time and guidance.

The requirements made by the Academic Editor

ABSTRACT:

Requirement 1: The abstract is excessively lengthy and should be limited to approximately 300 words. It is recommended to condense the content while retaining key findings and essential information.

Our response: We apologize for the lengthy abstract and have followed your advice to condense the content while retaining key findings and essential information. The abstract is now shortened and consists of 292 words. The revised version of the whole abstract has been added to the manuscript (lines 21-57).

Requirement 2: In the abstract, please specify the age range and characteristics of the adolescent participants along with the clinical setting.

Our response: Thank you for this reminder. We have now added the following information in the Methods part of the abstract: A total of 810 adolescents aged 11–15 years (392 boys, 418 girls) at Kanti Children’s Hospital in Kathmandu, were included. (lines 30-31).

Requirement 3: Clarify who provided which data.

Our response: We have clarified who provided which data in the Methods part: Adolescents reported past-year exposure to violence using adapted PedHITSS and ICAST-C items, while parents provided socio-demographic and family data. (lines 31-33).

Requirement 4: Briefly describe the questionnaire used, whether standardized or adapted tools, statistical software and analytical approach

Our response: Thank you for your suggestion. We have added the following, informing about the instruments used, analytical approach, and statistical software: “Adolescents reported past-year violence exposure using a modified version of PedHITSS and ICAST-C items, while parents provided socio-demographic and family data. Associations were examined using ordinal and logistic regression in SPSS 29.” (lines 31-34).

Requirement 5: Instead of stating “several socio-demographic and familiar factors” please explicitly mention the key variables that showed significant associations.

Our response: Thank you for pointing this out. We have now explicitly mentioned the key variables that showed significant associations, see the revised Results part of the abstract (lines 36-51).

Requirement 6: Provide appropriate statistical estimates (odds ratios, confidence intervals, p- values) for the findings.

Our response: As requested, we have added the appropriate statistical estimates for all findings throughout the revised Results part of the abstract (lines 36-51).

Requirement 7: The phrase “higher odds” appears frequently, you may consider using varied expressions such as “increased likelihood” or “greater risk”

Our response: We have now varied expressions according to your advice, using expressions like: “was more frequent” and “linked to increased…”, see the revised Results part of the abstract (lines 36-51)

Requirement 8: Consider streamlining the presentation for better readability

Our response: We have streamlined the presentation of the abstract for better readability – see the totally revised abstract version (line 21-56).

Requirement 9: Begin the Conclusion part with a clear summary of key findings based on your objective followed with concise implications.

Our response: Thank you for your suggestion. We have now revised the Conclusion part by providing a clear summary of key findings, followed by implications of our findings (lines 52-57).

BACKGROUND:

Requirement 10: Please incorporate regarding the perception of “child abuse” and “neglect” in Nepal context as well. The contextual scenario has been missed in justification of the research.

Our response: Thank you for this important comment. In response, we have added a paragraph to the Introduction that focuses on context and highlights cultural / contextual influences on the perception of child abuse and neglect, with particular emphasis on the Nepali context (lines 73-90).

Requirement 11: The statement “Gaining deeper understanding of this population is particularly urgent” needs more clarification. What aspect has been intended to explain here?

Our response: Thank you for your comment. We have reformulated the sentence and clarified the meaning as follows: “Such knowledge is particularly important in a context where mental health resources are scarce, and early intervention systems remain underdeveloped” (lines 114-116).

METHODS

Requirement 11: Participants: Justify the recruitment of this age group (11-15 years). As the study was conducted in CAP-OPD, the information regarding “patients were admitted to the hospital irrespective of their caste or ethnic background” is confusing.

Our response: We appreciate your comment and agree that our choice of age group requires clarification. Child and adolescent psychiatric outpatient services typically serve individuals from early childhood up to 16–18 years, with some variation across settings. However, due to hospital regulations at the study site (Kanti Children’s Hospital), children above 15 years are not admitted. We therefore included the oldest eligible group (11–15 years), although the broader age group (11–18 years) would have been preferable. As our data on maltreatment were based on self-reports, we focused on adolescents, as individuals aged 11–15 years are generally considered to have the cognitive and linguistic maturity to reliably complete self-administered screening instruments. We have added this information in the revised manuscript (lines 131-134).

To avoid confusion, we have moved the information about the hospital admitting children from all ethnic groups to the previous paragraph (“Study Site and Population”) (lines 123-126). In the following paragraph (“Participants”), we specify that participants were categorized based on caste and ethnicity for the purposes of this study (lines 143-152).

Requirement 12: Sample size calculation: How did you calculate the sample size and mention its adequacy.

Our response: The adequacy of the sample size will vary for different research questions depending on what the smallest meaningful effect size(s) would be, the measurement level of the dependent variable, and to some extent the amount of information and variation in both outcome variables and predictors. Unfortunately, we did not consider all power analytic aspects, but rather collected an as large as possible sample on the basis of a one-year time-frame We have added the following sentence in the Participants section: “The sample size was primarily determined by the fixed one-year data collection period at the clinic, during which 810 eligible participants were enrolled consecutively.” (lines 140-142).

Requirement 13: Did you take written consent from the child as well?

Our response: These sentences have been added at the onset of the Procedure section of the manuscript: “Before data collection, all participating adolescents and parents were informed about the project, both orally and in written form. Written, informed consent was obtained from all participants, i.e., both adolescents and their parents” (lines 155-157).

Requirement 14: How long did it take to complete the questionnaire?

Our response: Filling in the instruments about maltreatment took approximately 20-30 minutes. However, adolescents were not given a time limit to complete them. The following sentence has now been added to the manuscript: “Completing the maltreatment questionnaires took approximately 20–30 minutes. However, adolescents were not given a fixed time limit to complete them.” (lines 162-164)

Requirement 15: Which method of data collection was used? Any unexpected occurring aroused or not? Such as emotional break up?

Our response: Thank you for advising us to add this information. The following sentences have been added to the manuscript: “Data was collected using a self-administered, paper-based questionnaire completed by adolescents, while socio-demographic and family data were provided by one parent (either mother or father) and recorded manually by staff. Data collection was carried out by two office secretaries and one research assistant, all of whom received training, supervision, and monitoring from the first author throughout the data collection period. Completing the maltreatment questionnaires took approximately 20–30 minutes. However, adolescents were not given a fixed time limit to complete them. No unexpected incidents (e.g., emotional distress) were observed during the procedure” (lines 157-165).

Requirement 16: How the researchers assured the validity of the collected data as both parents were there even if collected differently.

Our response: Thank you for your comments. We agree that additional information is needed to clarify this. We have revised the manuscript, adding the following paragraph to clarify the procedure: To assure the validity of the sensitive data provided by the adolescents, the forms were completed by each adolescent alone in a separate, quiet room, prior to a clinical consultation, and with trained secretaries available to answer any queries and to clarify possible misun¬derstandings. Subsequently, the clinicians responsible reviewed the filled-in forms that the participants brought with them to the consultation room. Data plotting was done manually throughout the collection period” (lines 165-171).

Requirement 17: How were the instruments translated? Which translation process was used?

Our response: Thank you for this reminder. We have now made the information more precise. The following sentences have been included at the end of the Instruments section: “All instruments were translated into Nepali and then back translated by bilingual clinicians and professional translators to ensure accuracy and cultural relevance. The translation of tools was done with the standard procedure suggested by the WHO.” (line 205 - 207).

Requirement 18: Validity and reliability of the questionnaire?

Our response: Thank you for your suggestion. We agree that this information should be included. The following text on the validity and reliability of the PedHITSS questionnaire has been added to the manuscript (indicated here in bold): “This 5-item questionnaire was originally designed to detect and prompt provider investigation into child abuse in clinical settings and has been validated against the Conflict Tactics Scale: Parent-Child Version (CTSPC) demonstrating good internal consistency (α = .85) and strong concurrent validity (rₛ = .70, p < .01) (lines 182-183)

Requirement 19: It seems that there occurred clinical and legal issues requiring attention. Please elaborate more that because of this research participants did not get hurt and no harm was done.

Our response: Thank you for highlighting this important matter. Additional sentences have been added to elaborate more: “The study adhered to the ethical principle of non-maleficence, ensuring that no harm was done to any participant. Clinicians responsible for each patient reviewed all completed forms. Any cases of abuse identified through the screening process that required clinical intervention or legal action were promptly managed by the attending clinicians in accordance with established institutional protocols, and in coordination with the principal researcher. Thus, adolescent who needed future help were referred to the One Stop Crisis Management Center (OCMC) at Kanti Children’s Hospital and, where deemed appropriate, to the Child Workers in Nepal Concerned Centre (CWIN-Nepal) helpline, protection services and other needed services. This approach ensured participant safety while maintaining the integrity of the research procedures.” (lines 230-239)

Requirement 20: For those participants who frequently experienced sexual abuse, physical abuse, neglect what was the researchers’ role on it?

Our response: The researcher's role was to conduct the assessments about child abuse and neglect. Next, the patients moved on to meet with the clinicians responsible, taking with them the filled-in forms for the clinician to see. Identified participants with higher frequency of child maltreatment were referred by the clinicians to the One Stop Crisis Management Center (OCMC) at Kanti Children’s Hospital and, where deemed appropriate, to the Child Workers in Nepal Concerned Centre (CWIN-Nepal) helpline, protection services and other needed services.

DISCUSSION

Requirement 21: Do not just include the higher odds. Discuss your findings with meaningful interpretation highlighting the contextual scenario.

Our response: Thank you for this advice. We have reformulated the information about the results in the Discussion section (for Caste/Ethnicity, lines 412-413; for Gender, lines 427-433; for Family Structure, lines 474-477; and for Parental Education, lines 494-498). Contextual scenario is provided for all findings. Finally, we have added a few lines about the consequences of limited access to child protection services for marginalized groups to clarify meanings (lines 423-425),

Requirement 22: How do you explain the limited sample size?

Our response: Thank you for raising your concern. The following sentence about the limited sample size has been added in the revised manuscript under “Participants”:

The sample size was primarily determined by the fixed one-year data collection period at the clinic, during which 810 eligible participants were enrolled consecutively. (140-142).

In the “Limitations” of the Discussion, we have further elaborated:

Also, the relatively small and specific sample may have introduced selection bias, limiting the extent to which the findings can be generalized to the broader clinical adolescent population. Access to outpatient mental health services in Nepal is influenced by factors such as family socioeconomic status, parental education, and mental health awareness. Adolescents from families with greater resources or higher self-efficacy may be more likely to seek and obtain care, meaning that the present sample may not fully represent all adolescents with mental health problems in the clinical population. (lines 520-530)

Further, in response to your question, we would like to add that when we considered using power analysis to estimate sample size, we thought that a power analysis that just focuses on one of the research questions, or a strategy to collect data only within a relatively short time-frame were not optimal to secure enough power for each research question in the study.

Requirement 23: Also, the setting of this study reflects the tertiary referral hospital thus, consider these as well when writing limitation.

Our response: In response to your suggestion, we have included the following text in the “Limitations” of the Discussion section: “We acknowledge that the findings from a hospital-based child and adolescent psychiatric OPD may not be fully generalizable to non-clinical populations or to adolescents in other settings or regions” (lines 521-523).

CONCLUSIONS

Requirement 24: Mention clearly what are the key findings of this study first as of the objective. In the current writing, it is not known how the findings underscore an urgent need for targeted interventions.

Our response: Thank you for your comment. We have revised the Conclusions section to present the key findings and conclusions at the outset, in line with the study objectives and results. We have also removed the state

Attachment Submitted filename: Response to Reviewers.docx

# Editor report: Decision Letter 1

**DOI:** 10.1371/journal.pone.0355947.r003

**Authors:** Bimala Panthee

25 Jun 2026

Dear Dr. Sah,

Thank you for submitting your manuscript to PLOS ONE. After careful consideration, we feel that it has merit but does not fully meet PLOS ONE’s publication criteria as it currently stands. Therefore, we invite you to submit a revised version of the manuscript that addresses the points raised during the review process.

Most of the comments have been adequately addressed; however, there is still room for further improvement. The following suggestions are provided to enhance the overall quality and clarity of the manuscript:

Abstract

Background: The background section is concise; however, it would benefit from additional context regarding the importance of violence exposure and the existing gaps in the literature. Consider including a brief statement that more clearly aligns with the study title and strengthens the rationale for the research. In addition, the term “maltreatment” may not fully encompass the broader concept of “violence exposure.” Using “violence exposure” consistently throughout the abstract and manuscript would better reflect the study title, methodology, and overall focus of the research.

Introduction: The authors have provided relevant contextual information; however, similar to the concern raised for the abstract, the terminology requires greater clarity. The background should first introduce the broader concept of **violence exposure**  and then discuss specific forms such as abuse and maltreatment. Currently, the text appears to equate violence exposure with maltreatment, which may lead to conceptual ambiguity, as maltreatment represents only one aspect of violence exposure. Therefore, it is recommended that the authors introduce violence exposure as the overarching construct and subsequently position maltreatment within this broader framework, rather than beginning the discussion directly with maltreatment.

Participants: Please specify when the data collection period was.?: Line 132-133 “Eligible participants were adolescents aged 11–15 years who attended the unit during this period” is not clear.

It is suggested to rewrite this sentence “As data on maltreatment were obtained through self-reports, only adolescents were selected as informants, given their cognitive and linguistic maturity to reliably complete self-administered screening instruments.” to make it more academic writing.

Sample size: Thank you for the clarification provided in the response letter. However, this explanation should also be incorporated into the manuscript to ensure reader's understanding. In addition, the concern regarding sample size adequacy has not been fully resolved. Please provide a clear justification, as outlined in the response letter, including the number of outcome events and the number of predictors included in the models. This information is essential for readers to appropriately assess the robustness and validity of the study findings.

Limitation: The information mentioned in the limitation section regarding a relatively small sample does not justify this study’s limitation. Please consider your sample size with the number of your predictors so that the sample size will not be a limitation.

Line 155-157: Written informed consent from both adolescents and parents, or ascent from adolescents? Please check

Line 205-207: “All instruments were translated into Nepali and then back-translated by bilingual clinicians and professional translators to ensure accuracy and cultural relevance. The translation of tools was done with the standard procedure suggested by the WHO”. Citation required

Please submit your revised manuscript by Aug 09 2026 11:59PM. If you will need more time than this to complete your revisions, please reply to this message or contact the journal office at plosone@plos.org. When you're ready to submit your revision, log on to https://www.editorialmanager.com/pone/ and select the 'Submissions Needing Revision' folder to locate your manuscript file.

- A letter that responds to each point raised by the academic editor and reviewer(s). You should upload this letter as a separate file labeled 'Response to Reviewers'.
- A marked-up copy of your manuscript that highlights changes made to the original version. You should upload this as a separate file labeled 'Revised Manuscript with Track Changes'.
- An unmarked version of your revised paper without tracked changes. You should upload this as a separate file labeled 'Manuscript'.

If you would like to make changes to your financial disclosure, please include your updated statement in your cover letter. Guidelines for resubmitting your figure files are available below the reviewer comments at the end of this letter.

If applicable, we recommend that you deposit your laboratory protocols in protocols.io to enhance the reproducibility of your results. Protocols.io assigns your protocol its own identifier (DOI) so that it can be cited independently in the future. For instructions see: https://journals.plos.org/plosone/s/submission-guidelines#loc-laboratory-protocols. Additionally, PLOS ONE offers an option for publishing peer-reviewed Lab Protocol articles, which describe protocols hosted on protocols.io. Read more information on sharing protocols at https://plos.org/protocols?utm_medium=editorial-email&utm_source=authorletters&utm_campaign=protocols.

As the corresponding author, your ORCID iD is verified in the submission system and will appear in the published article. PLOS supports the use of ORCID, and we encourage all coauthors to register for an ORCID iD and use it as well. Please encourage your coauthors to verify their ORCID iD within the submission system before final acceptance, as unverified ORCID iDs will not appear in the published article. *Only*  the individual author can complete the verification step; PLOS staff *cannot*  verify ORCID iDs on behalf of authors.

We look forward to receiving your revised manuscript.

Kind regards,

Bimala Panthee

Academic Editor

PLOS One

Journal Requirements:

If the reviewer comments include a recommendation to cite specific previously published works, please review and evaluate these publications to determine whether they are relevant and should be cited. There is no requirement to cite these works unless the editor has indicated otherwise.

Please review your reference list to ensure that it is complete and correct. If you have cited papers that have been retracted, please include the rationale for doing so in the manuscript text, or remove these references and replace them with relevant current references. Any changes to the reference list should be mentioned in the rebuttal letter that accompanies your revised manuscript. If you need to cite a retracted article, indicate the article’s retracted status in the References list and also include a citation and full reference for the retraction notice.

[Note: HTML markup is below. Please do not edit.]

**********

[NOTE: If reviewer comments were submitted as an attachment file, they will be attached to this email and accessible via the submission site. Please log into your account, locate the manuscript record, and check for the action link "View Attachments". If this link does not appear, there are no attachment files.]

To ensure your figures meet our technical requirements, please review our figure guidelines: https://journals.plos.org/plosone/s/figures

You may also use PLOS’s free figure tool, NAAS, to help you prepare publication quality figures: https://journals.plos.org/plosone/s/figures#loc-tools-for-figure-preparation.

NAAS will assess whether your figures meet our technical requirements by comparing each figure against our figure specifications.

# Author comment: Author response to Decision Letter 2

**DOI:** 10.1371/journal.pone.0355947.r004

23 Jul 2026

Manuscript ID: PONE-D-25-60069

Title- Violence Exposure Among Adolescent Psychiatric Patients in Nepal: Socio-Demographic and Familial Risk Factors

Response to the reviewers

Thank you for your letter dated 26th June 2026 and for the thoughtful and constructive comments regarding our manuscript entitled Violence Exposure Among Adolescent Psychiatric Patients in Nepal: Socio-Demographic and Familial Risk Factors. Your feedback has helped us to improve the quality of our paper and we have carefully addressed all comments raised by the Academic Editor, as requested. Below is our point by point response to each concern raised. All changes have been highlighted / tracked in the revised manuscript file submitted herewith.

In addition to the revisions requested by the reviewers, we identified minor inaccuracies in the frequencies and percentages reported in Table 2 and in the corresponding text. These have been corrected. To improve consistency with the analytical approach, we also revised the table heading to: “Distribution of violence exposure outcome variables included in the regression analyses (n = 714).” (lines 314-341) for Table 2 and “Distribution of socio-demographic and family-related predictor variables included in the regression analyses (n=714)” for Table 1(lines 287-290, 304-305).

We hope that these additional revisions can be accepted by the journal and that all revisions made to the manuscript adequately address the remaining concerns. We believe the manuscript is now substantially strengthened and we hope it meets the journal's standards for publication. Thank you again for your time and guidance.

The requirements made by the Academic Editor

Abstract

Requirement 1:

Background:

The background section is concise; however, it would benefit from additional context regarding the importance of violence exposure and the existing gaps in literature. Consider including a brief statement that more clearly aligns with the study title and strengthens the rationale for the research. In addition, the term “maltreatment” may not fully encompass the broader concept of “violence exposure.” Using “violence exposure” consistently throughout the abstract and manuscript would better reflect the study title, methodology, and overall focus of the research.

Our response: Thank you for this feedback which strengthens the rationale of the study and clarifies the concept used for this study (child violence). We have now revised the background part of the abstract, providing additional context by addressing both the importance of the topic and existing gaps in literature. The text now reads:

“Violence exposure is a major public health concern among adolescents. Yet, evidence on socio-demographic and familial risk factors in adolescent psychiatric populations in Nepal and other low- and middle-income countries is limited”. (lines 23- 26)

Further, we have now changed the word “maltreatment” to “violence exposure” in accordance with your suggestion, both in the abstract (line 54) and also throughout the manuscript.

Requirement 2:

Introduction:

The authors have provided relevant contextual information; however, similar to the concern raised for the abstract, terminology requires greater clarity. The background should first introduce the broader concept of violence exposure and then discuss specific forms such as abuse and maltreatment. Currently, the text appears to equate violence exposure with maltreatment, which may lead to conceptual ambiguity, as maltreatment represents only one aspect of violence exposure. Therefore, it is recommended that the authors introduce violence exposure as the overarching construct and subsequently position maltreatment within this broader framework, rather than beginning the discussion directly with maltreatment.

Our response: Thank you for noting that the terminology that we use requires greater clarity, we quite agree that this is needed. We have now changed the overarching concept that we use, from “maltreatment” to “violence exposure”. Further, we have added a sentence early in the introduction of the manuscript to define the concept of child violence which includes maltreatment as one of its components. The sentence runs as follows:

“Violence exposure encompasses a broad range of experiences, including child maltreatment (physical, emotional, and sexual abuse and neglect), witnessing domestic or community violence, peer victimization and bullying, and other forms of interpersonal violence [1]” (lines-69-72)

Requirement 3:

Participants: Please specify when the data collection period was.?: Line 132-133 “Eligible participants were adolescents aged 11–15 years who attended the unit during this period” is not clear.

Our response: Thank you for pointing this out. The following sentence has now been added for clarification:

“The study population comprised adolescents aged 11-15 years during the data collection period 15 January 2023 to 15 January 2024.” (lines 136-137)

Requirement 4:

It is suggested to rewrite this sentence “As data on maltreatment were obtained through self-reports, only adolescents were selected as informants, given their cognitive and linguistic maturity to reliably complete self-administered screening instruments.” to make it more academic writing.

Our response: Thank you for your comment. We have added the following paragraph to make it more academic writing: “Only adolescents were selected as informants, given their cognitive and linguistic maturity to reliably complete self-administered screening instruments. Adolescents with diagnoses such as intellectual developmental disorder (IDD), acute psychosis, or any other condition that could compromise their cognitive and linguistic capacity to reliably complete self-administered maltreatment screening instruments, were excluded” (lines 137-142)

Requirement 5:

Sample size:

Thank you for the clarification provided in the response letter. However, this explanation should also be incorporated into the manuscript to ensure reader's understanding. In addition, the concern regarding sample size adequacy has not been fully resolved. Please provide a clear justification, as outlined in the response letter, including the number of outcome events and the number of predictors included in the models. This information is essential for readers to appropriately assess the robustness and validity of the study findings.

Our response: We thank you for highlighting the need for a clearer explanation of sample size adequacy. We have now made the needed explanation for the readers within the Statistical Analysis section of the manuscript under the subheading: “Sample adequacy”. The new text reads as follows:

“The sample size was primarily determined by the fixed one-year data collection period at the clinic, during which 810 eligible participants were enrolled consecutively. Of the 810 participants enrolled in the study, 31 were excluded due to missing data, and an additional 65 participants were excluded from the predictor analyses due to their classification in the "other" ethnicity category, which comprised a heterogeneous group. This exclusion reduced model complexity and allowed for a more clearly defined ethnicity variable. Consequently, the predictor analyses were conducted on a final analytical sample of 714 participants”. (lines 275-283).

We have also revised the manuscript to explicitly report the number of outcome events and the number of predictors included in the ordinal logistic regression models. We added the following paragraph:

“Our regression models included nine predictor variables (15 estimated parameters, excluding threshold parameters). Events-per-parameter (EPP) criterion for sample size adequacy states that approximately 10 events per estimated parameter is the conventional recommendation [36]. EPP values below this threshold may reduce the precision and stability of odds ratio estimates and increase the risk of overfitting [37]. The present study had the smallest number of cumulative events involved in the ordinal regression contained 83 cases for physical abuse, 32 for sexual abuse, 148 for emotional abuse, 173 for neglect and 66 for domestic violence, corresponding to EPP values ranging from approximately 2.1 to 11.5.” (lines 265-274)

Requirement 6:

Limitation section:

The information mentioned in the limitation section regarding a relatively small sample does not justify this study’s limitation. Please consider your sample size with the number of your predictors so that the sample size will not be a limitation.

Our response: Thank you for your kind suggestion to revise the limitation section of the Discussion chapter. We acknowledge that several models did not achieve the conventional EPP benchmark of 10 recommended by Peduzzi et al. Consequently, we cannot claim that the sample size was fully adequate for all regression analyses, particularly for outcomes with relatively few severe cases, such as sexual abuse and domestic violence. To ensure transparency, we have now explicitly acknowledged this limitation in the manuscript and noted that these findings should be interpreted with appropriate caution because limited EPP may reduce the precision and stability of the estimated associations and increase the risk of overfitting, as discussed by Peduzzi et al. (1996) and Babyak (2004).

The following information has now been added to the limitation section to explicitly detail our strategy to ensure model stability and robustness, and including also information on EPP ratios:

Moreover, several ordinal logistic regression models had EPP ratios below the conventional recommendation of 10, which may have reduced the precision and stability of odds ratio estimates and increased overfitting risk. Accordingly, these findings should be interpreted with caution.” (lines 548-551)

Requirement 7:

Line 155-157: Written informed consent from both adolescents and parents, or ascent from adolescents? Please check

Our response: We are sorry for mixing up. Now it has been corrected as follows:

“Written assent was obtained from all adolescents, and written informed consent was obtained from their parents, legal guardians or caregivers.” (lines 163-164).

Requirement 8:

Line 205-207: “All instruments were translated into Nepali and then back-translated by bilingual clinicians and professional translators to ensure accuracy and cultural relevance. The translation of tools was done with the standard procedure suggested by the WHO”. Citation required.

Our response: Thank you. Now we have added reference no 34 (line 215).

Attachment Submitted filename: Response to editor 2.docx

# Editor report: Decision Letter 2

**DOI:** 10.1371/journal.pone.0355947.r005

**Authors:** Bimala Panthee

29 Jul 2026

Violence Exposure Among Adolescent Psychiatric Patients in Nepal: Socio-Demographic and Familial Risk Factors

PONE-D-25-60069R2

Dear Dr. Sah,

We’re pleased to inform you that your manuscript has been judged scientifically suitable for publication and will be formally accepted for publication once it meets all outstanding technical requirements.

Within one week, you’ll receive an e-mail detailing the required amendments. When these have been addressed, you’ll receive a formal acceptance letter and your manuscript will be scheduled for publication.

An invoice will be generated when your article is formally accepted. Please note, if your institution has a publishing partnership with PLOS and your article meets the relevant criteria, all or part of your publication costs will be covered. Please make sure your user information is up-to-date by logging into Editorial Manager at Editorial Manager® and clicking the ‘Update My Information' link at the top of the page. For questions related to billing, please contact billing support.

If your institution or institutions have a press office, please notify them about your upcoming paper to help maximize its impact. If they’ll be preparing press materials, please inform our press team as soon as possible -- no later than 48 hours after receiving the formal acceptance. Your manuscript will remain under strict press embargo until 2 pm Eastern Time on the date of publication. For more information, please contact onepress@plos.org.

Kind regards,

Bimala Panthee

Academic Editor

PLOS One

Additional Editor Comments (optional):

Reviewers' comments:

# Editor report: Acceptance letter

**DOI:** 10.1371/journal.pone.0355947.r006

**Authors:** Bimala Panthee

PONE-D-25-60069R2

PLOS One

Dear Dr. Sah,

I'm pleased to inform you that your manuscript has been deemed suitable for publication in PLOS One. Congratulations! Your manuscript is now being handed over to our production team.

At this stage, our production department will prepare your paper for publication. This includes ensuring the following:

* All references, tables, and figures are properly cited

* All relevant supporting information is included in the manuscript submission,

* There are no issues that prevent the paper from being properly typeset

You will receive further instructions from the production team, including instructions on how to review your proof when it is ready. Please keep in mind that we are working through a large volume of accepted articles, so please give us a few days to review your paper and let you know the next and final steps.

Lastly, if your institution or institutions have a press office, please let them know about your upcoming paper now to help maximize its impact. If they'll be preparing press materials, please inform our press team within the next 48 hours. Your manuscript will remain under strict press embargo until 2 pm Eastern Time on the date of publication. For more information, please contact onepress@plos.org.

You will receive an invoice from PLOS for your publication fee after your manuscript has reached the completed accept phase. If you receive an email requesting payment before acceptance or for any other service, this may be a phishing scheme. Learn how to identify phishing emails and protect your accounts at https://explore.plos.org/phishing.

If we can help with anything else, please email us at customercare@plos.org.

Thank you for submitting your work to PLOS ONE and supporting open access.

Kind regards,

PLOS ONE Editorial Office Staff

on behalf of

Dr. Bimala Panthee

Academic Editor

PLOS One
